# Sociodemographic Determinants of Physical Inactivity of People Aged 60 Years and Older: A Cross-Sectional Study in Poland

**DOI:** 10.1155/2020/7469021

**Published:** 2020-12-10

**Authors:** Elżbieta Biernat, Monika Piątkowska

**Affiliations:** ^1^Collegium of World Economy, SGH Warsaw School of Economics, Warsaw, Poland; Al. Niepodległości 162, 02-554 Warsaw, Poland; ^2^Faculty of Physical Education, Josef Pilsudski University of Physical Education in Warsaw, Warsaw, Poland; 34 Marymoncka, 00-968 Warsaw, Poland

## Abstract

**Purpose:**

The aim of the study was to evaluate general physical activity (PA) level on the basis of leisure time and transportation physical activity (LTPA and TPA), assess the percentage of persons not meeting PA recommendations by the World Health Organisation (WHO), and evaluate the relationship between selected sociodemographic factors and physical inactivity.

**Methods:**

The paper is based on data (*n* = 7,347) retrieved from five large-scale surveys (2014-2018) used to collect information on the PA of Polish society. In order to meet the aim of the paper, we selected a sample of 2,023 Poles aged ≥ 60 years old. In each wave, the Polish long version of the International Physical Activity Questionnaire was used. Mann–Whitney *U* and Kruskal-Wallis tests were used to investigate the differences between the types and volume of PA and sociodemographic variables. Relationships between physical inactivity and analysed variables were evaluated using log-linear analysis. To capture relationships between physical inactivity and a set of explanatory variables, a predictive model was built.

**Results:**

The total average energy expenditure amounted to 1879.5 ± 2352.5 MET-min/week, including LTPA (938.5 ± 1491.9 MET-min/week) and TPA (944.8 ± 1322.4 MET-min/week). Over the course of the last two years of the study, the average value of MET-min/week increased significantly (*p* < 0.05); however, prohealth WHO norms are not met by nearly 40% of Poles. Sex determines the volume of LTPA and TPA (*p* < 0.05) but does not determine the inactivity of seniors. Place of residence and education differentiate participation in LTPA and TPA. The lower the education level and the smaller the place of residence, the greater the inactivity.

**Conclusions:**

The target for future interventions should be people aged 60+ living in villages and small towns (especially those with primary education). It is necessary to undertake educational and motivational programmes promoting PA. It is essential to develop detailed recommendations and to create a friendly and supportive environment.

## 1. Introduction

Physical inactivity is regarded as the fourth (behind high blood pressure, tobacco use, and high blood glucose) leading factor in the risk of global mortality [1]. This problem is observed particularly in Europe, where an ageing population and long-term growth of the costs of health care are serious health challenges [2]. Many of the leading causes of illnesses, such as coronary heart disease, cancer, and type 2 diabetes, could be counteracted by increasing the number of physically-active people among those who are inactive [3]. In the case of older people, this can bring very substantial health and social advantages. It has been proved that physical activity (PA) can decrease the drop in functional status of these people, enabling them to continue everyday activities by retarding functional limitations, hindering a drop in cognitive functions, and providing space for social interactions [4, 5]. The quality of their lives can be improved by improving physical, cognitive, and emotional functioning [5, 6]. Studies show that regular physical activity can decrease the risk of cognitive functions deteriorating by up to 38% [7], the risk of functional limitations by 30-50% [8, 9], and the risk of fractures of the femoral neck by 36-68% [8]. Finally, PA can retard the processes of dementia [8]. In the case of older people without dementia, exercise can significantly improve their memory, concentration, and mental processing speed [10], while those suffering from dementia can experience positive effects on their basic everyday life activities and physical functioning [11].

Due to the increasing costs of health and social care and the impact that the loss of autonomy can have on mental wellbeing, it is essential that seniors are independent for as long as possible [2], especially due to their increasing number in society. In Poland, at the end of 2016, the number of people of postworking age (men aged 65+ and women aged 60+) amounted to 7,770,000, which exceeded 1/5 of the total population [12]. The number of persons aged 60-64 has increased by nearly a half since 1989 [13]. Not many Poles in early old age (60-69) achieve the minimal dose of PA recommended by the World Health Organisation (WHO). According to a survey by Kantar TNS S.A. [14], these recommendations are realised by only 8% of respondents aged 60-69, while according to PolSenior [15]—one-third of those aged ≥ 65 (33.6%). Data by Eurobarometer [16] show that sport or leisure-time physical activity (LTPA) is completed regularly by just 8% of Poles aged 55-69 and 5% of those aged ≥ 70, while 33% and 30%, respectively, with some regularity. The Polish Central Statistical Office (GUS) states that only one-quarter of Poles aged 60+ (25.1%) participates in sports activities or physical recreation (regularly/often—10.6%; occasionally—14.5%) [17]. Transportation physical activity (TPA) is declared by 7% [18].

It is disheartening to observe that Polish seniors rarely participated in PA before retirement and continue the same pattern. Studies by Biernat et al. [19] show that a certain percentage of men stops exercising after retirement, which all means that the inactivity of today's population of older people will result in growing economic costs in the future. This is a substantial problem for public healthcare policy and the national economy [1].

Thus, it is very important to recognise the determinants of inactivity in these people. On the basis of previous Polish representative research, we know that men aged ≥ 60 are slightly more active (in general—27.7% and regularly/often—10.7%) than women (23.2% and 10.9%, respectively) [17]. Rowiński et al. [15] claim that there are no significant differences in the regular undertaking of LTPA between seniors living in villages and cities (39.6% and 39.9%, respectively). Single seniors less often participate in PA than those who are married [20].

Recommendations by the *American College of Sports Medicine* (ACSM) regarding health dose of PA are realised more often by men (40.9%) than women (29.2%), and considering the period of professional activity—more often blue-collar workers (33.6%), white-collar workers (37.8%), and others (33.2%) than farmers (25.3%) [15]. There are no significant differences in the realisation of recommended norms between seniors living in villages (35.4%) and cities below 500,000 citizens (31.4−41.3%, depending on the number of citizens) [15]. However, they are observed differences between respondents living in villages (35.4%) and cities over 500,000 citizens (27.2%).

It should also be mentioned that the studies described focus mainly on analyses of determinants of undertaking PA. However, in modern strategies of increasing PA/reversing the trend of its decrease, an indispensable element of the success is focusing on inactive persons. Considering knowledge about the conditioning of their physical passivity in new programmes can explain many issues and support recovery activities, which are a great challenge in our ageing society [21]. Thus, the aim of this work is to recognise a relationship between sociodemographic variables such as age, sex, place of residence, education level, and a lack of undertaking LTPA and TPA by Poles of early old age (60+). According to Mynarski et al. [22], LTPA and TPA are generally the only occasion for prohealth physical activity, especially among seniors.

The study considers three key areas: (1) evaluation of the overall level of PA on the basis of practiced LTPA and TPA; (2) assessment of the percentage of persons not meeting recommendations of optimal for health dose of PA by WHO; (3) evaluation of a real relationship between selected sociodemographic factors and physical inactivity of the population under study.

Cross-sectional research was carried out annually (from 2014 to 2018) on a random group of over 1000 people. As far as we know, this was the first time such a study, over such a time period and with such a large population, was conducted in Poland. The results may significantly enrich current knowledge about the participation of older adult Europeans in PA, broadening the area of international diagnosis by Poland, which is perceived as a socially and economically substantial state of East-Central Europe.

## 2. Material and Methods

### 2.1. Data Collection

The paper is based on data (*n* = 7,347) retrieved from five large-scale surveys (2014-2018) used to collect information on the PA of Polish society. All surveys were conducted by order of the Ministry of Sport and Tourism of the Republic of Poland. In each wave, the sample was random-quote and selected from the sampling frame of the National Official Register of the Territorial Division of the Country (TERYT). The sampling procedure included three stages: territorial stratification, drawing addresses, and allocation of demographic characteristics. Computer-Assisted Personal Interviews (CAPI) were conducted by trained and supervised pollsters. The ethics committee of the Polish Academy of Sciences approved the study (approval nr. KEwN/60/2014) in accordance with the Declaration of Helsinki (2004). Participation was voluntary and confidential, and informed consent was obtained from participants before completing the survey.

In each wave, the same standardized questionnaire—the Polish long version of the International Physical Activity Questionnaire (IPAQ-LF) [23]—was used. IPAQ-LF provides information about the frequency, duration, and intensity of activities during the previous seven days. The questionnaire assesses four domains in which PA is performed: leisure time, domestic, occupational, and transportation. In this study, only questions on LTPA (including vigorous PA (VPA), moderate PA (MPA), and walking) and TPA (including cycling and walking) were analysed. The minimum duration of a single PA is set at 10 minutes.

### 2.2. Participants

The data were gathered on representative samples of Poles aged 15-69 in 2014 (*n* = 1,019), 2015 (*n* = 1,020), 2016 (*n* = 2,118), 2017 (*n* = 2,131), and 2018 (*n* = 1,059). Out of the whole sample (*n* = 7,347), we selected people aged 60 years or older. Therefore, the sample comprised 1,996 Poles (837 males and 1,159 females). Subjects who did not declare the presence of diseases were eligible for the study. Sex, level of education (primary, vocational, secondary, and higher), and place of residence (village, towns up to 500,000 inhabitants, and towns with 500,000 inhabitants) were considered as sociodemographic indicators for descriptive analysis.  Table 1 provides descriptive statistics for the whole sample.

### 2.3. Data Analysis

Data were prepared and analysed according to IPAQ guidelines for data processing and analysis [24]. From the initial sample (*n* = 2,023), cases with missing data (*n* = 17) and “unreasonably high” values (reports of activity in excess of 16 hours/day considered implausible) (*n* = 10) were removed from the study (*n* = 27). A final sample (*n* = 1,996) was used for further analysis.

On the basis of the duration (min/day) and frequency (days/week) of the specific activities, TPA and LTPA (expressed in MET-min/week) of the group were calculated. A weekly energy expenditure of the activity was calculated by multiplying a MET number attributed to it (VPA—8 MET, MPA—4 MET, walking—3.3 MET, and cycling—6 MET) by the number of days of practising it per week and time of duration in minutes per day. 1 MET corresponds to the consumption of O_2_ during rest and equals 3.5 ml O_2_/kg of body mass per minute [25]. Meeting WHO recommendations meant undertaking moderate for ≥150 minutes/week or vigorous PA for ≥75 minute/week or walking for ≥150 minutes/week, or an equivalent of a combination of all activities (LTPA, TPA) exceeding 600 MET-minute/week [1].

### 2.4. Statistical Analysis

The data collected by the questionnaires were analysed using IBM® SPSS® Statistics ver. 22. A descriptive analysis was performed to explore the sample characteristics (frequencies and percentages), and PA levels (means—$\;\overset{¯}{x}$) and standard deviations (±SD) were calculated using the IPAQ-LF.

In order to verify if analysed variables (LTPA: VPA, MPA, and walking and TPA: cycling and walking) were characterized by a normal distribution, the Kolmogorov-Smirnov test was used for a single sample. Due to not meeting the above assumption for dependent variables (*p* < 0.05), the statistical inference was based on nonparametric tests. Mann–Whitney *U* and Kruskal-Wallis tests were used to investigate the differences between the types and volume of PA and sociodemographic variables.

Relationships between physical inactivity (LTPA and TPA) and analysed sociodemographic criteria were evaluated using log-linear analysis. The strength of this relationship was expressed by the odds ratio (OR) with a 95% confidence interval.

In order to capture relationships between a categorical dependent variable (taking up LTPA and TPA was the target variable) and a set of explanatory variables (sex, education, and place of residence), a predictive model—Chi-squared automatic interaction detection (CHAID) decision tree, was built. The Chi-squared test and maximum likelihood classification were used to compare different categorical variables, which were classified into a binary or more series by the most significant predictor. The significance level for node-splitting of the decision tree in CHAID was set at *α* = 0.05. The estimated error of risk in the model was 0.403, and the standard error was 0.011.

Statistical significance was set at *α* = 0.05 for all analyses. The data PDF is provided in supplementary materials in the Editorial's repository (see available here).

## 3. Results

The conducted analysis revealed that in the studied group of Poles aged ≥ 60, 78.9% undertake some amount of PA (LTPA or TPA), while 21.1% are completely inactive in this area.

Among those declaring participation in PA, the total average energy expenditure amounted to 1879.5 ± 2352.5(1054.5) MET-min/week– including LTPA (938.5 ± 1491.9(938.5) MET-min/week) and TPA (944.8 ± 1322.4(396.0) MET-min/week). Whereas significant differences were noted between men and women (*p* = 0.008) in their declarations of undertaking LPTA (LTPA only, without TPA) for the benefit of males (1054.9 ± 1574.1(438.0) and 854.6 ± 1424.4(346.5) MET-min/week, respectively), especially when it comes to recreational walking (*p* = 0.018; 685.6 ± 1077.6(198.0) and 540.7 ± 908.8(99.0) MET-min/week, respectively). It was similar in the case of TPA, transportation cycling in particular. Men (237.9 ± 751.7(0) MET-min/week) chose this type of transport more often (*p* = 0.0001) than women (119.1 ± 488.5(0) MET-min/week).

An essential importance in undertaking LTPA (*p* = 0.001) and TPA (*p* = 0.031) was noticed also in the aspect of the level of education. The higher the education level, the greater the average MET-min/week of energy expenditure coming from VPA and MPA (Table 2). In the field of TPA, persons with secondary education were more active (1039.2 ± 1440.1(462.0) MET-min/week) than those with primary education (844.7 ± 1230.0(292.5) MET-min/week), basic/vocational education (940.7 ± 1312.0(396.0) MET-min/week), and higher education (938.4 ± 1191.0(414.0) MET-min/week).

The place of residence was also a variable differentiating LTPA and TPA of persons in this study. A significantly higher (*p* = 0.0001) energy expenditure resulting from LTPA was observed among inhabitants of large cities (over 500,000 citizens) in comparison with other respondents, i.e., those living in villages and cities below 500,000 citizens (Table 2). TPA was higher (*p* = 0.002) among seniors living in cities below 500,000 citizens. However, in the case of particular forms of TPA, these correlations were different. For example, transport by bicycle was more often (*p* = 0.006) declared by inhabitants of villages (204.3 ± 627.1(0) MET-min/week) and walking by those living in cities over 500,000 citizens (*p* = 0.0001; 840.6 ± 1096.3(330.0) MET-min/week).

The analysis of LTPA and TPA revealed that WHO prohealth norms were met by 61.1% of respondents, which means that nearly 40% of Poles aged 60+ in the years 2014-2018 did not undertake such physical activity that would keep up their health.

Verification of determinants that can have a relationship with physical inactivity showed that sex does not condition a lack of LTPA and TPA (Table 3). However, the level of education and place of residence of seniors correlate with them. The higher education, the less common is lack of LTPA. Persons with basic vocational education (*p* = 0.09; 39.3%), secondary education (*p* = 0.001; 33.2%), and higher education (*p* = 0.001; 23.8%) declared lack of participation in LTPA significantly less often (only LTPA, without TPA) than respondents with primary education (43.3%). The smaller the place of residence, the more often the physical inactivity. Lack of LTPA was more often declared by respondents living in villages (46.6%) than those living in cities (below 500 000—*p* = 0.001; 34.0% and over 500 000—*p* = 0.001; 32.4%).

The level of education and place of residence proved also to be essential conditions for not undertaking TPA (Table 3). In the former, significant differences were observed between persons with primary education (41.9%) and secondary education (*p* = 0.003; 33.4%) or higher education (*p* = 0.007; 30.6%). In the latter, lack of TPA was more often declared by people living in villages (43.1%) in comparison with those from cities below 500 000 (*p* = 0.001; 34.5%) and over 500 000 (*p* = 0.001; 32.4%).

**Figure 1:**
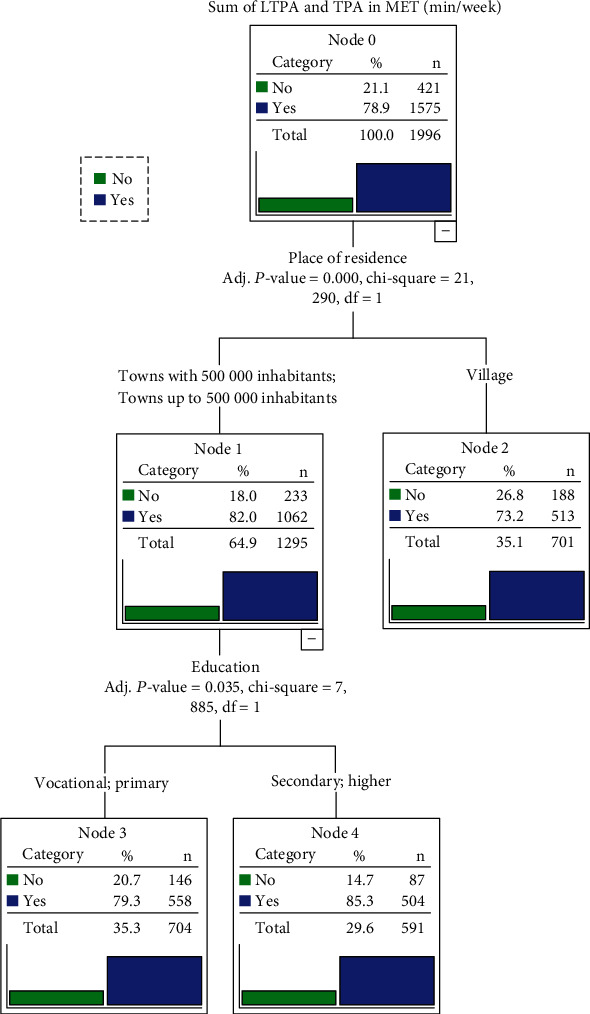
A CHAID decision classification tree analysis to identify the risk factors of physical inactivity. Abbreviations: CHAID: Chi-squared Automatic Interaction Detector; LTPA: leisure-time physical activity; TPA: transport-related physical activity; adj. *p* value: adjusted level of significance; df: degrees of freedom.

In accordance with the CHAID decision classification tree analysis, the most essential determinant of inactivity of older Poles is the place of residence (Figure 1). Lack of LTPA and TPA (jointly) was more often (*p* = 0.001) noted among respondents living in villages (26.2%) and smaller cities (below 500 000 citizens—18.0%) than among those living in cities with over 500 000 citizens (14.2%). Inactive persons living in cities below 500 000 inhabitants are mainly those with primary and basic vocational education (20.7%).

## 4. Discussion

As mentioned at the beginning of the article, this study is the first major representative research conducted in a period of several years (2014-2018), analysing determinants of lack of PA among Poles aged over 60. PA (LTPA and TPA) was considered as, in general, it is the only possibility of undertaking prohealth physical activity in case of these persons [22].

The results revealed that although during the last two years of the study the average value of MET-min/week of physical activities undertaken by respondents within LTPA and TPA significantly increased, many seniors still remain inactive. Over 21% of them do not undertake any PA during leisure time (either LTPA or TPA), while nearly 40% do not realise the prohealth dose of PA recommended by WHO, which is necessary to maintain good health condition. This may result in illnesses characteristic for this age group, as well as harmful negative social and economic effects for the whole society. According to *The economic cost of physical inactivity in Europe* [2], in Poland, lack of PA results in annual direct costs of healthcare to the sum of EUR 219 million (due to the influence of PA on the four main health problems: developing cancer, type II diabetes, heart disease, and suffering from premature death). Inactivity of Poles also generates an additional 1.3 billion EURO of indirect costs, calculated on the basis of the estimated economic value of a healthy life lost due to illnesses and premature death. Depression and anxiety (also related with lack of PA) result in indirect economic costs amounting to 658 million EURO [2]. Consequences of this type depend on the behaviour of seniors [26]. Biernat and Piątkowska [27] prove that WHO recommendations are not met by Poles in the last three phases of life: professionally active 50-64-year old childless persons (46.1%), professionally inactive 50-64-year-old childless persons (50.3%), and childless pensioners aged 65+ (47.6%). Other Polish studies [19] show that going into retirement (in Poland, 60 years for women and 65 for men) has a quite significant importance in this scope, as at the moment of losing contact with the work environment, the PA of seniors visibly decreases.

The calculations of economists show that Polish society can avoid these costs entirely by encouraging inactive persons to introduce simple changes in their lifestyles, such as including physical exercise in everyday or weekly activities. If we manage to encourage half of currently physically inactive Poles to exercise or practise sport, savings on public healthcare would amount to about 440 million PLN a year [26]. This sum is close to the annual budget expenditure of an average-size city in Poland. However, even decreasing the number of persons not achieving the recommended level of PA itself would bring advantages. According to *The economic cost of physical inactivity in Europe* [2], increasing the 20% fraction of people not meeting PA norms recommended by WHO can result in advantages for Poland to the amount of 435 million EURO.

All of this indicates the importance of activating the oldest group of Poles, 1/5 of Polish society, who are most vulnerable to illnesses. Our studies show that it is essential to implement activities that stimulate and support changes in their lifestyle. It is necessary to develop strategies, programmes, and particular recommendations. However, such activities should be based on the most up-to-date knowledge about changing determinants of inactivity. For example, previous studies suggested that sex is an important determinant of PA. As a result, more attention was paid to women who were less often active than men [28–30]. Currently, since the mid-1990s, women have successively decreased the difference in participation in LTPA compared to men [31, 32]. Our results show that sex conditions the volume of undertaken LTPA and TPA (more often participation of men); however, it does not determine the inactivity of seniors. Place of residence and education have a significant importance for participation in LTPA and TPA, as well as lack of activity. Lack of LTPA and TPA is more often observed among people living in villages and smaller cities (below 500 000 citizens) than in those living in cities over 500 000 inhabitants, in the groups living in cities below 500 000, and more often among those with primary and basic vocational education. In the case of participation, the situation is the opposite—the higher the education level and the larger the place of residence, the higher the level of LTPA.

The phenomenon that people living in larger cities are more active and those from villages and smaller cities less often participate in LTPA is not rare in the EU [33]. Being too far from activity facilities, lower social support, and fewer pavements are major barriers for living in rural areas [34, 35]. However, an example from the Netherlands shows that it is possible to cause more common participation in LTPA of people living in rural areas [30], by taking care of their physical environment (climate, presence of natural elements, space for sport, etc.) [30] and building a friendly social environment (e.g., safety in districts) [36]. Obviously, it is much easier in the Netherlands, where the majority of people are of a higher social and economic status. However, in Poland, where people of a higher professional, economic, and educational status move from large agglomerations to suburbs, the social environment can also undergo changes. Such changes can result in new patterns of behaviour, including awareness of the necessity of taking care of one's own health. Currently, the lack of LTPA and TPA among seniors living in small cities (below 500 000 citizens) is observed mainly among those with primary and basic vocational education. This indicates poor awareness of the role of physical activity in a long and healthy life. People change their behaviour when they are aware of its unhealthy results [37]. When they are not aware of this, they do not see the need to change and do not undertake any activities [38]. What is more, they even hide behind a subjectively evaluated state of health and perception of harmfulness of PA to their illnesses (especially older people) [39].

Lack of PA is not solely a consequence of personal problems or an unfriendly environment, but also, to large extent, a result of choices made [40]. It may seem that there are no obstacles to walking (apart from real functional limitations) [41]. However, our study shows that over one-third of persons aged 60+ do not undertake any form of active transportation (on foot or by cycling). Of course, we are aware of the correlation between age and decreased active transportation, which results, unfortunately, in a greater risk of various illnesses, including being overweight and obesity [42, 43]. In the case of Poland, this concerns persons with primary education in particular (41.9%). Lack of TPA in this group is significantly more common compared to persons with secondary (33.4%) and higher education (30.6%). This validates the assumption about lack of knowledge of the importance of PA in maintaining good health. This is also indicated by other Polish reports [44], which state that lack of awareness is especially visible when analysing physical inactivity among those with low education and financial constraints. Unfortunately, in rural and small-town environments, current health education faces numerous barriers related to lack of appreciation of the importance of health prophylaxis, lack of infrastructure for its promotion, insufficient activity of self-governments in this field, and a lack of personal role models and qualified promoters of health [34, 45]. Thus, it seems necessary to intensify these activities and to implement programmes promoting walking or cycling. Apart from gardening [46], these are the forms of PA for older people recommended by WHO [1].

## 5. Conclusions

The aim of this study was to specify the level of leisure-time physical activity of Poles aged 60+ and to determine what factors condition lack of LTPA and TPA. The results revealed that although during the last two years of the study, the average value of MET-min/week of physical activities undertaken by respondents within LTPA and TPA significantly increased, nearly 40% of Poles do not meet prohealth norms set by WHO.

Sex determines the volume of LTPA and TPA but does not determine the inactivity of seniors. Place of residence and education differentiate participation in LTPA and TPA. Thus, seniors from rural areas and small towns, including those with primary education, are a suitable target group for future interventions. It is necessary to address them with educational programmes that increase their awareness concerning the prohealth role of PA. In this scope, it is essential to prepare detailed recommendations about the duration of PA, its form, and intensity (in Poland, national norms for high-risk groups have not so far been developed) [47]. The process of developing such norms can be a starting point for including the issue of the PA of seniors in the national agenda.

It is also essential to motivate older people to be physically active and to create a supportive environment (including for walking and cycling). One example of efficient intervention can be promoting the use of pedometers [48], building cycle paths, or designing footpaths on which stations that propose exercises would be situated (including explanations of how they can be helpful). Older people must experience prohealth advantages in order to believe in regular PA and include it in their daily routine [49]. Other helpful items would be information boards promoting additional physical activity, such as to use stairs instead of a lift. It is also necessary to facilitate access to sports infrastructure [50, 51] and to change the character and image of such places. Currently, older persons do not regard themselves as addressees of such facilities. They regard them as natural forms of organisation, but mainly for young people [27, 52].

## 6. Strengths and Weakness of the Study

A strong side of this study is the representativeness of sample and PA analysis (LTPA and TPA) of persons aged 60+ over a longer period of time (2014–2018). Due to the selection and size of the analysed group (*n* = 1996), the results and conclusions for the analysed sample can be extended for the whole population. However, the study embraces a limited number of analysed determinants. Considering the essence of the problem (not only economic, but also social), the implementation of the process of active ageing, defined by the *Organisation for Economic Co-operation and Development* (OECD) as one's capability to lead a productive life in the society and economy in the course of ageing, requires a further analysis of complementary factors in the lives of seniors [53].

## Figures and Tables

**Table 1:** Characteristics of the sample.

Factors		*N*	%
Sex	Male	837	41.9
	Female	1159	58.1

Level of education	Primary	499	25.0
	Basic vocational	765	38.3
	Secondary	572	28.7
	Higher	160	8.0

Place of residence	Village	702	35.2
	Towns up to 500 000 inhabitants	1042	52.2
	Towns with 500 000 inhabitants	252	12.6

Year of the study	2014	271	13.6
	2015	276	13.8
	2016	564	28.3
	2017	585	29.3
	2018	300	15.0

Total		1996	100.0

**Table 2:** LTPA (VPA, MPA, and walking) and TPA (cycling and walking) in MET-min/week declared by respondents in 2014-2018 ($\bar{\;x}\;$and ±SD).

Variables		LTPA (*N* = 1996)				TPA (*N* = 1996)		
		VPA	MPA	Walking	Total	Cycling	Walking	Total
Sex	Male (*N* = 837)	168.7 ± 712.8	199.7 ± 559.4	685.6 ± 1077.6	1054.9 ± 1574.1	237.8 ± 751.7	816.5 ± 1135.6	1054.9 ± 1489.8
	Female (*N* = 1159)	122.6 ± 648.7	190.8 ± 581.8	540.6 ± 908.8^a^	854.6 ± 1424.4^a^	119.1 ± 488.5^a^	746.0 ± 1050.3	865.3 ± 1181.3

Level of education	Primary (*N* = 499)	138.3 ± 693.9	185.1 ± 606.0^c^	540.2 ± 960.9^bc^	864.3 ± 1523.9^bc^	131.8 ± 448.3	712.6 ± 1069.6^bc^	844.7 ± 1230.0^bc^
	Vocational (*N* = 765)	153.2 ± 707.9	184.5 ± 556.5^e^	597.2 ± 996.5	936.3 ± 1525.2^de^	169.1 ± 576.8	770.9 ± 1092.1	940.7 ± 1312.0
	Secondary (*N* = 572)	128.4 ± 632.8	202.8 ± 568.6	662.3 ± 1027.8	993.5 ± 1461.9	213.5 ± 805.8	825.6 ± 1107.2	1039.2 ± 1440.1
	Higher (*N* = 160)	147.9 ± 622.1	242.2 ± 555.5	593.8 ± 838.0	983.9 ± 1331.7	123.8 ± 422.2	814.5 ± 1044.1	938.4 ± 1191.0

Place of residence	Village (*N* = 702)	105.9 ± 557.4^f^	171.0 ± 553.7	472.0 ± 906.5^f^	749.6 ± 1322.5^f^	204.3 ± 627.1	668.8 ± 1069.9^f^	873.7 ± 1328.9
	Towns up to 500 000 inhabitants (*N* = 1042)	143.4 ± 657.6^g^	204.1 ± 586.9	658.4 ± 2021.2^g^	1005.9 ± 1515.7^g^	156.3 ± 605.6	831.5 ± 1092.1	987.8 ± 1331.8
	Towns with 500 000 inhabitants (*N* = 252)	235.9 ± 980.7	220.5 ± 653.0	725.0 ± 1010.5	1185.1 ± 1759.9	122.7 ± 620.0	840.6 ± 1096.3	964.2 ± 1260.1

Year	2014 (*N* = 271)	58.6 ± 451.0	116.3 ± 353.9^i^	529.6 ± 905.7^ijk^	704.5 ± 1180.0^jk^	95.1 ± 390.5	525.4 ± 903.9^jk^	620.5 ± 1005.9^jk^
	2015 (*N* = 276)	92.2 ± 591.3	69.1 ± 257.4^l^	466.6 ± 788.8^mn^	627.8 ± 1123.0^mn^	103.3 ± 416.3	452.2 ± 673.3^mn^	555.5 ± 825.0^mn^
	2016 (*N* = 564)	192.4 ± 654.1	361.2 ± 734.5^op^	260.3 ± 606.9^op^	813.9 ± 1192.9^op^	172.8 ± 605.2	433.1 ± 748.5^o^	605.9 ± 1 − 19.6^op^
	2017 (*N* = 585)	135.3 ± 750.9	147.2 ± 563.1	872.4 ± 1153.2	1154.9 ± 1753.7	190.0 ± 711.4	1086.9 ± 1241.1^r^	1276.9 ± 1520.6^r^
	2018 (*N* = 300)	181.2 ± 791.5	159.5 ± 546.8	907.2 ± 1184.8	1252.5 ± 1845.4	247.3 ± 732.0	1343.2 ± 1339.1	1593.9 ± 1606.6

Total	*N* = 1996	142.0 ± 676.5	194.5 ± 572.4	601.4 ± 985.4	938.5 ± 1491.9	168.9 ± 615.4	775.5 ± 1087.2	944.8 ± 1322.4

Abbreviations: $\bar{\;x}$: mean; ±SD: standard deviation; LTPA: leisure-time physical activity; VPA: vigorous physical activity; MPA: moderate physical activity; TPA: transport-related physical activity; ^a-f^ statistically significant *p* < 0.05, ^a^—women vs. men, ^b^—primary vs. secondary, ^c^—primary vs. higher, ^d^—vocational vs. secondary, ^e^—vocational vs. higher, ^f^—village vs. towns up to 500 000 inhabitants, ^g^—towns up to 500 000 inhabitants vs. towns with 500 000 inhabitants, ^h^—2014 vs. 2015, ^i^—2014 vs. 2016, ^j^—2014 vs. 2017, ^k^—2014 vs. 2018, ^l^—2015 vs. 2016, ^m^—2015 vs. 2017, ^n^—2015 vs. 2018, ^o^—2016 vs. 2017, ^p^—2016 vs. 2018, ^r^—2017 vs. 2018.

**Table 3:** Odds ratio of no TPA and LTPA (with the 95% confidence interval) by respondents.

Variables		Total physical inactivity			No LTPA			No TPA		
		*N* (%)	OR (95% CI)	*p*	*N* (%)	OR (95% CI)	*p*	*N* (%)	OR (95% CI)	*p*
Sex	Male	163 (19.5)	—	—	296 (35.4)	—	—	308 (36.8)	—	—
	Female	258 (22.3)	0.84 (0.68-1.05)	0.132	449 (38.7)	0.87 (0.72-1.04)	0.124	436 (37.6)	0.97 (0.8-1.16)	0.708

Level of education	Primary	133 (26.7)	—	—	216 (43.3)	—	—	209 (41.9)-	—	—
	Basic vocational	165 (21.6)	1.32 (1.02-1.72)	0.022	301 (39.3)	1.18 (0.94-1.48)	0.091	295 (38.6)	1.45 (0.91-1.45)	0.131
	Secondary	100 (17.5)	1.72 (1.28-2.23)	0.001	190 (33.2)	1.54 (1.12-1.97)	0.001	191 (33.4)	1.44 (1.12-1.84)	0.003
	Higher	23 (14.4)	2.17 (1.33-3.51)	0.001	38 (23.8)	2.45 (1.64-3.67)	0.001	49 (30.6)	1.63 (1.12-2.39)	0.007

Place of residence	Village	188 (26.8)	—	—	327 (46.6)	—		302 (43.1)		—
	Towns up to 500 000 inhabitants	197 (18.9)	1.57 (1.25-1.97)	0.001	354 (34.0)	1.7 (1.4-2.07)	0.001	360 (34.5)	1.43 (1.18-1.75)	0.001
	Towns with 500 000 inhabitants	36 (14.2)	2.21 (1.49-3.26)	0.001	64 (32.4)	2.58 (1.88-3.56)	0.001	82 (32.4)	1.58 (1.17-2.14)	0.002

Total		421 (21.1)			745 (37.3)			744 (37.3)		

Abbreviations: LTPA: leisure-time physical activity; TPA: transport-related physical activity; OR: odds ratio; CI: confidence interval; *p* value: level of significance.

## Data Availability

The data used to support the findings of this study are available from the corresponding author upon request.
