# Supplementary material for: Sociodemographic Determinants of Physical Inactivity of People Aged 60 Years and Older: A Cross-Sectional Study in Poland
Source: Biomed Res Int. 2020 Dec 10;2020:7469021. doi: 10.1155/2020/7469021 (PMC7787744; doi:10.1155/2020/7469021)
Supplement: Supplementary Materials — The data PDF is provided in supplementary materials in the Editorial's repository. [file 7469021.f1.pdf]

NUMERANKIETYSexWiekWiek_katWiek15_29wiek_polStan_cywOSWGOSPosw2FAZAŻYCIAEdudochód1dochód2STATUSZAWODOWYstatus2stat_zaw_polKLASASPINTERNETInternet2MZAMIESZKANIAMzamplace_resAkt_

1674721	2	66	6		5	2		8	2	2	4	2	4	3	4	3
1674723	1	65	6		5	2		8	1	1	9	5	3	3	4	3
1674739	1	69	6		5	2		8	2	4	9	5	4	3	1	1
1674740	2	67	6		5	1		8	2	4	9	5	4	3	1	1
8	2			5	5	2		8	2	2	9	5	3	3	3	2
12	1			5	5	2		8	2	2	9	5	4	3	2	2
12	1			5	5	1		8	2	1	9	5	4	3	3	2
13	1			5	5	2		8	2	2	9	5	4	3	2	2
13	2			5	5	2		8	2	2	9	5	4	3	3	2
22	2			5	5	1		8	1	1	9	5	5	3	3	2
31	2			5	5	5		4	3	4	9	5	2	3	2	2
34	1			5	5	2		8	3	3	9	5	2	3	3	2
38	2			5	5	1		8	1	1	9	5	5	3	4	3
41	2			5	5	4		6	2	4	9	5	3	2	4	3
66	2			5	5	4		4	2	2	9	5	4	3	4	3
69	2			5	5	5		4	1	3	6	3	4	3	1	1
73	2			5	5	2		8	1	1	6	3	5	3	1	1
75	2			5	5	2		8	3	3	9	5	2	3	3	2
83	1			5	5	2		8	3	2	9	5	3	3	2	2
86	2			5	5	3		7	3	4	9	5	2	3	2	2
98	2			5	5	2		8	2	2	9	5	4	3	3	2
107	2			5	5	2		8	1	3	9	5	4	3	3	2
108	2			5	5	2		8	1	1	9	5	5	3	1	1
131	2			5	5	2		8	2	1	9	5	4	3	1	1
135	1			5	5	2		8	2	1	9	5	4	3	1	1
146	2			5	5	3		8	1	1	6	3	5	3	1	1
182	2			5	5	1		8	2	1	9	5	5	3	1	1
187	2			5	5	1		8	1	1	9	5	5	3	2	2
190	1			5	5	2		8	2	2	9	5	4	3	1	1
192	2			5	5	1		8	1	1	9	5	5	3	1	1
210	2			5	5	1		8	4	1	9	5	3	3	3	2
222	2			5	5	5		6	3	4	9	5	2	3	1	1
237	1			5	5	2		8	3	2	6	3	3	3	1	1
242	1			5	5	4		6	2	3	9	5	3	3	1	1
248	1			5	5	1		8	1	1	9	5	5	3	1	1
250	1			5	5	1		8	1	1	6	3	5	3	1	1
251	1			5	5	2		8	2	1	9	5	4	3	2	2
290	2			5	5	2	blad		1	1	6	3	5	3	1	1
291	2			5	5	2		8	1	1	6	3	5	3	1	1
300	2			5	5	2		8	3	3	9	5	3	3	2	2
301	1			5	5	2		7	3	3	9	5	2	2	2	2
305	1			5	5	2		7	3	4	2	1	2	1	2	2
327	1			5	5	2		7	2	3	6	3	3	3	1	1
334	2			5	5	2		8	1	2	9	5	4	2	2	2
334	1			5	5	2		8	2	1	9	5	4	3	2	2
341	2			5	5	5		6	1	1	7	4	5	3	2	2
344	2			5	5	1		8	1	1	9	5	5	3	1	1
358	1			5	5	1		8	2	1	9	5	4	3	1	1
358	2			5	5	3		8	1	2	6	3	4	3	1	1
375	1			5	5	2		8	2	2	9	5	4	3	3	2
377	1			5	5	1		8	1	1	9	5	5	3	3	2
381	2			5	5	1		8	3	1	9	5	3	3	3	2
387	2			5	5	1		8	4	1	9	5	3	3	3	2
387	2			5	5	1		8	4	1	9	5	3	2	4	3
400	2			5	5	5		5	1	2	9	5	4	3	2	2
405	2			5	5	2		8	2	3	9	5	3	3	2	2
405	2			5	5	3		8	1	2	6	3	4	3	2	2
412	2			5	5	2		8	3	4	7	4	1	3	4	3
414	2			5	5	5		4	1	1	6	3	5	2	1	1
424	2			5	5	2		8	1	2	9	5	4	3	1	1
432	1			5	5	2		8	1	3	9	5	4	3	1	1
436	2			5	5	5		5	2	4	9	5	2	3	1	1
440	2			5	5	2		8	3	3	9	5	3	3	2	2
441	2			5	5	2		8	1	2	6	3	5	3	1	1
443	1			5	5	3		8	1	1	9	5	5	3	1	1
446	2			5	5	2		8	3	3	9	5	3	3	1	1
449	2			5	5	1		8	1	1	9	5	5	3	1	1
481	1			5	5	2		7	4	4	1	1	1	1	4	3
482	2			5	5	2		8	1	2	6	3	4	3	1	1
491	1			5	5	5		4	2	3	4	2	3	2	3	2
504	2			5	5	2		8	1	2	6	3	5	3	1	1
505	2			5	5	1		8	1	1	6	3	5	3	1	1
506	2			5	5	1		8	1	1	6	3	5	3	1	1
508	1			5	5	3		8	3	3	9	5	3	3	1	1
514	2			5	5	2		8	3	2	9	5	3	2	2	2
516	2			5	5	1		8	3	1	9	5	3	2	2	2
539	1			5	5	1		8	2	1	9	5	4	3	4	3
540	2			5	5	3		8	3	2	9	5	3	3	1	1
545	1			5	5	2		8	3	4	9	5	1	3	3	2
573	1			5	5	2		8	2	4	9	5	3	3	4	3
576	2			5	5	1		8	3	1	9	5	3	3	3	2
583	1			5	5	2		7	2	3	6	3	3	2	1	1
584	2			5	5	2		8	2	2	6	3	4	3	1	1
599	1			5	5	1		8	2	1	9	5	4	3	3	2
603	2			5	5	2		8	2	2	9	5	4	3	1	1
604	1			5	5	2		8	1	2	9	5	4	3	1	1
609	1			5	5	2		8	1	2	9	5	4	3	1	1
610	1			5	5	2		8	2	2	9	5	4	1	3	2
615	1			5	5	1		8	2	1	9	5	4	3	3	2
616	2			5	5	3		8	3	2	9	5	3	3	2	2
620	1			5	5	4		7	1	3	9	5	4	3	1	1
621	2			5	5	2		7	1	2	9	5	5	3	1	1
632	1			5	5	2		8	3	2	9	5	3	3	2	2
641	1			5	5	1		8	2	1	9	5	4	3	1	1
645	2			5	5	3		8	2	2	9	5	4	3	1	1
647	1			5	5	2		8	2	3	9	5	3	3	1	1
656	1			5	5	2		7	2	2	4	2	4	3	1	1
658	2			5	5	2		8	2	2	9	5	4	2	1	1
682	1			5	5	1		8	1	1	9	5	5	3	2	2
684	2			5	5	4		8	1	2	6	3	4	3	1	1
687	1			5	5	3		8	2	3	9	5	3	3	1	1
691	1			5	5	2		8	2	2	6	3	4	3	1	1
695	2			5	5	2		8	1	1	6	3	5	3	1	1
697	1			5	5	3		8	1	1	9	5	5	3	1	1
699	2			5	5	1		8	1	1	6	3	5	3	1	1
706	2			5	5	2		8	1	1	6	3	5	3	1	1
717	1			5	5	2		7	3	2	1	1	2	1	2	2
724	1			5	5	2		8	1	1	9	5	5	3	2	2
725	2			5	5	2		5	2	1	9	5	5	3	2	2
735	2			5	5	1		8	3	2	9	5	3	3	3	2
746	2			5	5	5		4	2	3	6	3	3	3	1	1

762	1	5	5	1	8	4	3	9	5	2	3	2	2
762	1	5	5	2	8	3	2	9	5	3	3	2	2
767	2	5	5	1	8	3	1	9	5	3	3	3	2
777	2	5	5	3	8	1	3	6	3	4	3	1	1
778	1	5	5	2	8	1	2	6	3	4	3	1	1
784	2	5	5	2	7	3	3	2	1	2	1	1	1
785	2	5	5	2	8	3	2	6	3	3	3	2	2
797	2	5	5	2	8	1	1	9	5	5	3	3	2
809	2	5	5	2	8	3	2	7	4	2	2	2	2
815	2	5	5	1	8	1	1	6	3	5	3	2	2
817	2	5	5	1	8	1	1	9	5	5	3	2	2
822	2	5	5	5	4	4	4	9	5	1	1	1	1
824	2	5	5	2	8	1	1	6	3	5	3	1	1
840	2	5	5	2	8	1	2	6	3	5	3	1	1
841	2	5	5	2	8	1	2	6	3	4	3	1	1
854	2	5	5	1	8	3	1	9	5	3	3	2	2
863	1	5	5	1	8	2	1	9	5	5	3	3	2
865	2	5	5	2	6	1	1	9	5	5	3	3	2
873	2	5	5	1	8	3	1	9	5	3	3	3	2
883	1	5	5	2	7	2	1	9	5	5	3	2	2
887	2	5	5	2	8	1	2	6	3	4	3	1	1
901	1	5	5	2	8	2	2	9	5	4	3	1	1
902	1	5	5	2	8	2	2	9	5	4	3	1	1
908	2	5	5	2	8	1	1	6	3	5	3	1	1
909	1	5	5	2	8	1	1	6	3	5	3	1	1
916	2	5	5	4	6	1	4	6	3	3	3	1	1
924	2	5	5	2	8	3	3	9	5	3	3	1	1
926	2	5	5	2	8	3	3	9	5	3	3	2	2
929	2	5	5	3	7	2	3	9	5	3	3	2	2
935	2	5	5	2	7	2	2	4	2	4	3	2	2
952	2	5	5	2	8	3	2	9	5	3	3	1	1
957	1	5	5	2	8	2	1	9	5	4	3	1	1
961	2	5	5	2	8	3	1	9	5	3	3	1	1
964	2	5	5	5	4	1	2	6	3	4	3	1	1
974	2	5	5	2	8	1	1	6	3	5	3	1	1
989	2	5	5	2	7	1	2	6	3	5	3	1	1
1014	2	5	5	1	8	3	1	9	5	3	1	4	3
1029	2	5	5	2	8	4	4	9	5	1	1	1	1
1034	2	5	5	3	8	3	2	9	5	3	3	1	1
1042	2	5	5	1	8	1	1	9	5	5	3	1	1
1048	1	5	5	3	8	1	2	9	5	4	3	3	2
1050	2	5	5	1	8	3	1	9	5	3	3	3	2
1053	1	5	5	2	8	3	2	9	5	3	2	3	2
1061	2	5	5	2	8	3	2	9	5	3	3	3	2
1064	2	5	5	3	6	2	2	9	5	3	3	2	2
1068	1	5	5	2	7	2	3	4	2	3	3	2	2
1070	1	5	5	2	8	1	1	9	5	5	3	2	2
1072	1	5	5	3	7	2	3	9	5	3	3	3	2
1072	2	5	5	1	8	2	1	9	5	4	3	2	2
1076	2	5	5	4	6	2	3	9	5	3	3	3	2
1084	1	5	5	2	8	2	1	9	5	4	3	3	2
1093	2	5	5	2	8	2	1	9	5	4	3	3	2
1097	2	5	5	1	8	3	1	9	5	3	3	3	2
1110	2	5	5	2	8	1	2	9	5	5	3	2	2
1116	2	5	5	1	8	2	1	9	5	5	3	1	1
1117	2	5	5	2	8	1	2	6	3	4	3	1	1
1122	1	5	5	2	8	2	2	9	5	4	3	1	1
1129	1	5	5	2	7	3	1	9	5	4	3	3	2
1136	1	5	5	1	8	2	1	9	5	5	3	1	1
1144	2	5	5	1	8	3	1	9	5	3	3	4	3
1155	2	5	5	1	8	3	1	9	5	3	3	4	3
1156	1	5	5	2	8	4	3	9	5	2	1	4	3
1157	1	5	5	1	8	4	1	9	5	3	2	4	3
1200	1	5	5	2	8	3	1	9	5	3	3	3	2
1204	2	5	5	2	8	1	1	9	5	5	3	3	2
1204	2	5	5	2	7	3	2	7	4	3	3	1	1
1207	2	5	5	2	8	1	1	6	3	5	3	1	1
1227	2	5	5	3	8	3	2	9	5	3	3	2	2
1242	2	5	5	2	8	3	3	9	5	3	3	3	2
1252	1	5	5	2	8	2	1	6	3	4	3	3	2
1253	2	5	5	2	8	2	2	9	5	3	3	3	2
1255	2	5	5	2	8	3	3	9	5	2	3	3	2
1266	1	5	5	5	4	1	2	6	3	4	3	1	1
1271	2	5	5	2	8	1	1	6	3	5	3	1	1
1272	2	5	5	2	8	1	1	6	3	5	3	1	1
1282	1	5	5	2	7	3	3	2	1	2	2	3	2
1293	2	5	5	2	8	3	3	9	5	2	2	1	1
1321	1	5	5	2	7	4	3	4	2	3	1	3	2
1326	1	5	5	2	8	2	2	9	5	4	3	1	1
1329	2	5	5	4	8	2	3	9	5	3	3	1	1
1329	2	5	5	1	8	2	1	9	5	4	3	1	1
1330	1	5	5	2	7	2	3	9	5	3	3	1	1
1337	2	5	5	2	8	3	3	9	5	3	3	4	3
1350	2	5	5	3	7	3	4	9	5	2	3	2	2
1356	2	5	5	1	8	2	1	9	5	4	3	2	2
1360	1	5	5	1	8	2	1	6	3	5	3	1	1
1361	2	5	5	2	8	3	2	9	5	3	3	1	1
1371	2	5	5	2	8	2	3	9	5	3	3	2	2
1374	2	5	5	2	8	1	2	9	5	5	3	2	2
1379	2	5	5	2	8	1	2	9	5	5	3	2	2
1380	1	5	5	2	8	2	3	9	5	3	3	2	2
1381	1	5	5	2	8	2	3	9	5	4	3	2	2
1389	1	5	5	2	8	2	2	9	5	4	3	1	1
1392	2	5	5	1	8	1	1	9	5	5	3	1	1
1412	1	5	5	4	6	1	3	9	5	3	3	2	2
1418	2	5	5	2	8	4	3	9	5	2	3	1	1
1425	2	5	5	2	7	1	2	6	3	4	3	1	1
1426	2	5	5	2	8	1	2	6	3	5	3	1	1
1429	2	5	5	2	8	1	2	6	3	5	3	1	1
1439	2	5	5	3	8	1	3	6	3	4	3	1	1
1442	2	5	5	5	6	1	2	9	5	4	3	1	1
1449	2	5	5	3	6	2	1	9	5	4	1	3	2
1455	2	5	5	2	8	4	4	9	5	2	2	2	2
1461	2	5	5	3	8	2	2	9	5	4	3	2	2
1465	2	5	5	3	8	2	4	6	3	3	3	1	1
1470	1	5	5	1	7	2	2	4	2	4	3	1	1
1474	2	5	5	3	7	2	4	9	5	3	3	1	1
1491	1	5	5	2	8	3	1	6	3	3	3	3	2
1497	2	5	5	2	8	3	2	9	5	3	3	3	2
1498	1	5	5	1	8	1	1	9	5	5	3	3	2
1505	1	5	5	2	7	4	3	1	1	2	1	3	2
1508	2	5	5	5	4	1	1	6	3	5	3	1	1

1517	1		6		5	4		1		3		2		7	5			8		3	2
1519	2			5	5		2		8	2	1		6		3	5	3		1		1
1521	1			5	5		2		8	2	2		9		5	4	3		2		2
1534	2			5	5		2		8	4	3		9		5	2	3		1		1
1548	2			5	5		1		8	2	1		9		5	5	3		3		2
1571	2			5	5		1		8	2	1		9		5	5	3		4		3
1573	1			5	5		1		8	1	1		9		5	5	3		4		3
1576	2			5	5		2		8	2	2		6		3	4	3		1		1
1582	1			5	5		1		8	1	1		9		5	5	3		1		1
1590	2			5	5		1		8	1	1		9		5	5	3		1		1
1593	2			5	5		1		8	2	1		9		5	5	3		3		2
1602	2			5	5		5		6	1	2		9		5	5	3		2		2
1617	2			5	5		2		8	1	3		9		5	4	3		3		2
1619	2			5	5		1		8	3	1		9		5	3	2		4		3
1638	2			5	5		1		8	1	1		9		5	5	3		1		1
1639	2			5	5		2		8	3	1		9		5	4	3		2		2
1647	1			5	5		4		8	1	2		9		5	4	3		1		1
1656	2			5	5		1		8	3	1		9		5	3	3		2		2
1671	1			5	5		2		8	3	3		9		5	2	1		3		2
1678	2			5	5		1		8	1	1		9		5	5	3		1		1
1679	1			5	5		3		7	2	4		9		5	3	3		3		2
1686	1			5	5		3		8	1	3		9		5	3	3		1		1
1688	2			5	5		1		8	1	1		6		3	5	3		1		1
1708	1			5	5		2		8	2	3		9		5	3	3		3		2
1713	2			5	5		1		8	1	1		6		3	5	3		2		2
1724	2			5	5		3		6	1	1		9		5	5	3		2		2
1728	1			5	5		2		8	2	3		9		5	3	3		3		2
1730	2			5	5		2		8	2	2		9		5	4	3		3		2
1749	2			5	5		5		4	1	4		6		3	3	3		1		1
1752	1			5	5		2		8	2	1		6		3	4	3		1		1
1773	1			5	5		2		7	4	4		1		1	2	1		3		2
1787	2			5	5		1		8	1	1		6		3	5	3		1		1
1793	2			5	5		1		8	3	1		9		5	3	3		1		1
1795	1			5	5		2		8	3	3		9		5	2	3		3		2
1814	2			5	5		2		8	2	3		9		5	3	3		3		2
1826	1			5	5		2		8	2	2		9		5	4	3		1		1
1839	2			5	5		1		8	2	1		9		5	5	3		4		3
1841	2			5	5		1		8	1	1		6		3	5	3		1		1
1844	1			5	5		1		8	1	1		9		5	5	3		4		3
1847	2			5	5		3		8	2	2		9		5	4	3		1		1
1848	1			5	5		2		8	2	2		9		5	3	3		1		1
1851	2			5	5		1		8	3	1		9		5	3	3		1		1
1852	1			5	5		1		8	2	1		9		5	4	3		1		1
1857	2			5	5		2		8	2	2		9		5	4	3		1		1
1443747	2	76	6		5	4		1		1		1		7	5			8		1	1
1443747	2		6		5	4		1		2		1		7	5			8		1	1
1443767	1		6		5	2		1		2		3		7	5			8		1	1
1443785	1	60	6		5	2		3		2		4		7	5			8		1	1
1443786	1		6		5	2		5		2		4		7	5			8		1	1
1443789	2	61	6		5	4		4		2		4		7	5			8		1	1
1443798	1		6		5	2		2		2		4		7	5			8		1	1
1443801	2	60	6		5	2		2		3		3		4	2			8		1	1
1443812	1		6		5	1		1		1		1		9	7			8		1	1
1443818	2	63	6		5	2		2		4		4		7	5			2		1	1
1443824	2		6		5	2		2		1		2		7	5			8		1	1
1443826	1		6		5	4		1		1		1		7	5			8		1	1
1443835	1	62	6		5	2		2		3		4		7	5			1		1	1
1443843	1	66	6		5	2		2		1		4		7	5			8		1	1
1443846	2		6		5	4		3		2		4		7	5			8		1	1
1443855	1	68	6		5	4		1		2		4		7	5			8		1	1
1443856	2	67	6		5	4		1		1		4		7	5			8		1	1
1443872	2	77	6		5	4		1		1		1		7	5			8		1	1
1443873	1		6		5	2		3		2		4		7	5			8		1	1
1443881	1	70	6		5	2		2		4		4		7	5			1		1	1
1443881	1		6		5	2		4		1		3		7	5			8		1	1
1443893	1		6		5	4		1		2		4		7	5			8		1	1
1443894	2	62	6		5	2		2		2		4		7	5			8		1	1
1443897	2		6		5	4		1		2		1		7	5			8		1	1
1443902	1		6		5	2		1		2		2		4	2			8		1	1
1443906	2	70	6		5	4		1		2		4		7	5			8		1	1
1443917	2	73	6		5	2		1		1		1		7	5			8		1	1
1443919	1	67	6		5	2		1		2		4		7	5			8		1	1
1443935	2		6		5	2		2		3		3		3	1			1		1	1
1443936	2	76	6		5	2		2		3		4		7	5			8		1	1
1443944	1	79	6		5	2		2		3		2		7	5			8		1	1
1443952	2	62	6		5	4		1		3		2		7	5			1		1	1
1443955	1	63	6		5	2		3		3		3		7	5			8		1	1
1443957	2	61	6		5	2		2		2		2		7	5			8		1	1
1443964	1		6		5	4		1		3		4		7	5			8		1	1
1443966	2		6		5	4		1		4		4		7	5			8		1	1
1443969	2	68	6		5	2		2		1		3		7	5			8		1	1
1443970	1	71	6		5	2		2		1		4		7	5			8		1	1
1443972	2	61	6		5	4		2		3		2		7	5			8		1	1
1443973	1	73	6		5	2		2		2		2		4	2			8		1	1
1443974	1		6		5	2		3		2		3		4	2			8		1	1
1444005	1	68	6		5	2		2		1		4		7	5			8		1	1
1444005	1		6		5	2		3		3		2		7	5			8		1	1
1444006	2	63	6		5	4		4		1		3		7	5			8		1	1
1444006	2		6		5	2		5		1		4		7	5			8		1	1
1444007	2	70	6		5	4		1		1		1		7	5			8		1	1
1444008	1	69	6		5	2		2		1		4		7	5			8		1	1
1444022	1	61	6		5	2		2		2		3		7	5			8		1	1
1444025	1		6		5	2		2		2		4		7	5			8		1	1
1444026	2		6		5	4		1		3		4		7	5			8		1	1
1444027	2		6		5	4		5		2		4		7	5			8		1	1
1444028	1		6		5	2		2		1		2		7	5			8		1	1
1444029	2		6		5	4		5		1		4		7	5			8		1	1
1444034	2		6		5	2		2		3		4		7	5			1		1	1
1444035	1	60	6		5	3		1		2		1		7	5			8		1	1
1444039	2	70	6		5	4		2		1		2		7	5			8		1	1
1444042	2	67	6		5	2		2		3		4		7	5			4		1	1
1444049	2		6		5	1		1		3		3		2	1			8		1	1
1444051	2	77	6		5	4		1		1		1		7	5			8		1	1
1444066	1	65	6		5	2		3		3		3		4	2			8		1	1
1444071	2	66	6		5	4		1		2		2		7	5			8		1	1
1444071	2		6		5	2		2		2		2		7	5			8		1	1
1444087	2		6		5	4		3		1		4		7	5			8		1	1
1444091	2	65	6		5	2		2		2		4		7	5			8		1	1
1444097	1		6		5	2		2		2		2		9	7			8		1	1
1444104	2	61	6		5	4		1		1		1		7	5			1		1	1
1444111	1	76	6		5	4		1		1		4		7	5			8		1	1

1444122	1	72	6	5	2	2	4	4	7	5	8	1	1
1444123	1		6	5	2	2	2	4	7	5	2	1	1
1444137	2		6	5	2	2	2	4	7	5	8	1	1
1444139	2		6	5	2	2	1	4	7	5	8	1	1
1444145	1		6	5	2	3	1	4	7	5	8	1	1
1444148	2		6	5	4	1	1	4	7	5	8	1	1
1444148	2	80	6	5	4	1	1	1	7	5	8	1	1
1444149	1	60	6	5	2	2	1	4	7	5	8	1	1
1444155	1	64	6	5	3	1	2	1	4	2	8	1	1
1444156	2		6	5	2	2	3	4	4	2	8	1	1
1444162	2	65	6	5	2	2	2	3	7	5	8	2	2
1444162	1		6	5	2	2	2	3	4	2	8	1	1
1444163	2		6	5	4	1	1	1	7	5	8	1	1
1444169	2		6	5	2	2	1	2	7	5	8	1	1
1444171	1		6	5	2	4	1	4	7	5	8	1	1
1444172	2	70	6	5	4	1	1	1	7	5	8	2	2
1444175	2	61	6	5	2	2	3	3	3	1	2	2	2
1444181	2		6	5	4	1	2	1	7	5	8	2	2
1444186	2	61	6	5	4	1	3	1	7	5	2	2	2
1444186	1		6	5	2	2	2	3	4	2	8	2	2
1444195	2	66	6	5	2	2	2	4	7	5	8	2	2
1444203	2	62	6	5	3	2	2	3	7	5	8	2	2
1444208	2	80	6	5	2	2	1	2	7	5	8	2	2
1444209	1	76	6	5	4	1	2	3	7	5	8	2	2
1444231	1	80	6	5	4	1	2	4	7	5	8	2	2
1444232	2	72	6	5	4	1	1	4	7	5	8	2	2
1444233	2	65	6	5	2	2	2	3	7	5	8	2	2
1444234	1	66	6	5	2	2	3	3	4	2	1	2	2
1444235	1		6	5	2	2	2	2	7	5	8	2	2
1444236	2		6	5	2	2	2	2	6	4	8	2	2
1444263	1	66	6	5	2	2	2	3	7	5	1	2	2
1444268	2	80	6	5	4	1	1	4	7	5	8	2	2
1444277	2	64	6	5	2	2	2	4	7	5	8	2	2
1444277	1		6	5	2	2	2	4	7	5	8	2	2
1444282	1	66	6	5	4	2	3	4	7	5	8	2	2
1444293	2		6	5	2	4	2	4	7	5	8	2	2
1444296	2		6	5	2	2	3	4	7	5	8	2	2
1444297	2	75	6	5	2	4	1	3	7	5	8	2	2
1444297	1		6	5	1	1	1	4	9	7	8	2	2
1444300	1	62	6	5	2	3	1	3	7	5	1	2	2
1444302	2		6	5	2	3	3	3	7	5	8	2	2
1444312	2	71	6	5	4	1	2	2	7	5	8	3	2
1444312	1		6	5	2	2	2	3	7	5	8	2	2
1444313	1	68	6	5	2	2	2	3	7	5	8	3	2
1444315	2		6	5	4	1	3	4	7	5	1	2	2
1444318	1		6	5	2	2	2	4	7	5	8	2	2
1444319	2		6	5	2	2	3	4	7	5	1	2	2
1444336	1		6	5	2	2	2	3	7	5	8	2	2
1444339	2	61	6	5	2	2	1	4	9	7	8	3	2
1444351	1		6	5	2	2	4	4	1	1	1	2	2
1444352	1	77	6	5	2	2	1	4	7	5	8	3	2
1444357	2	78	6	5	4	1	3	4	7	5	8	3	2
1444376	2	68	6	5	2	3	1	3	7	5	8	3	2
1444382	2	61	6	5	4	1	2	2	7	5	8	3	2
1444383	1		6	5	2	2	2	4	7	5	8	3	2
1444389	2	60	6	5	3	1	3	2	4	2	1	3	2
1444390	1	64	6	5	2	2	3	4	7	5	8	3	2
1444395	2		6	5	4	1	1	4	7	5	8	3	2
1444397	1	68	6	5	2	3	2	3	7	5	8	3	2
1444398	2	72	6	5	4	2	1	3	7	5	8	3	2
1444398	1		6	5	2	2	2	3	7	5	8	3	2
1444407	2	71	6	5	4	2	2	3	7	5	8	3	2
1444409	2		6	5	2	3	3	3	7	5	4	3	2
1444411	2	66	6	5	2	3	2	3	4	2	8	3	2
1444420	1	67	6	5	2	2	2	4	7	5	8	3	2
1444421	2	67	6	5	2	2	2	3	7	5	8	3	2
1444423	1	61	6	5	2	2	2	3	7	5	8	3	2
1444432	2	68	6	5	4	1	2	1	7	5	8	3	2
1444433	1	64	6	5	2	2	2	3	7	5	8	3	2
1444439	2		6	5	2	3	4	3	1	1	1	3	2
1444445	2		6	5	3	1	2	2	7	5	2	3	2
1444449	1		6	5	2	2	2	3	7	5	8	3	2
1444461	1	64	6	5	2	3	3	3	7	5	2	3	2
1444467	1	68	6	5	1	1	3	2	7	5	8	3	2
1444469	2		6	5	2	2	3	4	7	5	1	3	2
1444473	2	67	6	5	4	1	2	2	7	5	8	3	2
1444477	1	62	6	5	3	1	2	2	7	5	1	3	2
1444478	2	66	6	5	4	1	2	2	7	5	8	3	2
1444493	2	76	6	5	2	2	1	4	7	5	8	3	2
1444495	1	61	6	5	1	1	2	4	9	7	8	3	2
1444496	2	77	6	5	1	1	1	1	7	5	8	3	2
1444503	2		6	5	2	1	3	4	4	2	8	3	2
1444505	1	61	6	5	1	2	2	4	7	5	8	3	2
1444506	2	75	6	5	4	1	2	4	7	5	8	3	2
1444507	2	78	6	5	4	2	2	2	7	5	8	4	2
1444508	1	74	6	5	2	2	3	3	7	5	8	4	2
1444509	1		6	5	2	2	3	3	4	2	4	3	2
1444510	2		6	5	2	2	3	2	7	5	1	3	2
1444515	1		6	5	2	2	2	3	7	5	8	3	2
1444519	2	64	6	5	3	1	2	1	7	5	8	4	2
1444525	1		6	5	4	1	3	2	7	5	8	3	2
1444526	2		6	5	2	2	3	2	7	5	8	3	2
1444530	2	63	6	5	4	1	3	2	7	5	8	4	2
1444530	2		6	5	2	3	2	3	7	5	8	3	2
1444531	1	84	6	5	2	2	3	4	7	5	8	4	2
1444531	1		6	5	4	1	2	3	7	5	8	3	2
1444540	2		6	5	2	3	4	2	7	5	8	3	2
1444543	1		6	5	2	2	3	4	7	5	8	3	2
1444544	2		6	5	2	3	3	1	7	5	8	3	2
1444546	1	62	6	5	3	1	2	1	7	5	8	4	2
1444555	2		6	5	2	2	3	3	7	5	8	4	2
1444556	1		6	5	2	2	3	4	7	5	8	4	2
1444562	1	62	6	5	3	1	2	2	7	5	8	4	2
1444563	2	62	6	5	4	1	2	2	7	5	8	4	2
1444566	2		6	5	3	2	3	3	7	5	8	4	2
1444567	1		6	5	2	2	2	3	7	5	8	4	2
1444574	2	65	6	5	2	2	2	3	7	5	8	4	2
1444577	1	65	6	5	2	2	3	4	4	2	8	4	2
1444579	2		6	5	4	1	3	4	7	5	8	4	2
1444587	2		6	5	4	1	4	4	7	5	8	4	2
1444599	1		6	5	3	1	3	1	7	5	1	4	2

1444617	1		6	5	4	1	2		2		7	5	8		4	2
1444618	2		6	5	2	2	2		3		7	5	8		4	2
1444623	1	72	6	5	2	2	2		3		7	5	8		4	2
1444631	2	62	6	5	1	3	4		4		3	1	1		4	2
1444632	1	60	6	5	3	1	4		2		3	1	1		4	2
1444639	2	88	6	5	4	1	1		1		7	5	8		4	2
1444642	1	65	6	5	4	1	2		2		7	5	8		4	2
1444643	2		6	5	3	1	3		4		7	5	8		4	2
1444645	2		6	5	3	4	3		4		7	5	8		4	2
1444656	2		6	5	2	2	3		4		2	1	1		4	2
1444662	2	66	6	5	4	1	1		1		7	5	8		4	2
1444664	2	72	6	5	4	1	4		1		7	5	8		4	2
1444671	2		6	5	3	3	1		3		7	5	2		4	2
1444672	2		6	5	3	1	3		1		1	1	1		4	2
1444679	2	60	6	5	2	3	1		4		7	5	8		4	2
1444680	1	76	6	5	2	2	2		4		7	5	8		4	2
1444681	2	63	6	5	3	1	4		4		9	7	8		4	2
1444681	1		6	5	3	1	1		1		7	5	8		4	2
1444690	1	60	6	5	2	2	3		3		4	2	8		4	2
1444692	2		6	5	2	1	3		4		7	5	8		4	2
1444693	1	77	6	5	4	1	1		4		7	5	8		5	3
1444702	2	77	6	5	2	2	1		2		7	5	8		5	3
1444704	2	71	6	5	2	2	1		4		7	5	8		5	3
1444707	2	81	6	5	4	2	1		4		7	5	8		5	3
1444714	2		6	5	2	2	2		3		4	2	8		4	2
1444726	2	69	6	5	4	1	2		4		7	5	8		5	3
1444732	2	69	6	5	4	1	3		2		7	5	8		5	3
1444737	2	62	6	5	2	3	4		4		7	5	8		5	3
1444741	1		6	5	2	2	3		3		7	5	8		4	2
1444743	2		6	5	2	2	2		2		6	4	8		4	2
1444747	2	61	6	5	4	1	2		4		7	5	8		5	3
1444752	1		6	5	2	2	2		4		7	5	2		4	2
1444753	2		6	5	2	2	3		4		7	5	1		4	2
1444754	2	62	6	5	2	2	2		4		7	5	8		5	3
1444754	2		6	5	4	1	1		1		7	5	8		5	3
1444755	1		6	5	4	1	4		4		1	1	1		5	3
1444766	2		6	5	4	2	1		3		7	5	8		5	3
1444767	2	65	6	5	2	2	3		3		3	1	1		5	3
1444768	1	60	6	5	2	1	3		3		3	1	1		5	3
1444780	2	66	6	5	2	2	3		3		7	5	2		5	3
1444785	1		6	5	4	4	3		3		7	5	8		5	3
1444788	1	62	6	5	2	2	4		3		4	2	1		5	3
1444797	2		6	5	4	2	1		4		7	5	8		5	3
1446171	2	63	6	5	2	2	2		4		7	5	8		2	2
1446171	2		6	5	2	2	3		3		3	1	4		5	3
1446172	2		6	5	4	1	4		2		7	5	2		5	3
1446173	1		6	5	2	2	4		3		3	1	1		5	3
1446192	2		6	5	4	1	3		2		7	5	1		5	3
1447055	2		6	5	2	2	3		3		7	5	8		5	3
1447507	1		6	5	2	2	3		3		3	1	1		5	3
1447509	2	68	6	5	4	1	2		1		7	5	8		3	2
1447510	1	66	6	5	2	2	2		2		7	5	8		3	2
1447566	1	60	6	5	2	2	2		3		7	5	1		1	1
1447578	2	60	6	5	2	2	4		4		3	1	1		4	2
1447898	1	64	6	5	2	2	2		4		7	5	8		1	1
1447898	1		6	5	2	2	2		3		7	5	8		1	1
1447902	2	75	6	5	2	2	2		2		7	5	8		1	1
1447923	2	74	6	5	4	2	1		4		7	5	8		1	1
1447925	2	61	6	5	3	4	3		4		7	5	8		3	2
1447926	2	60	6	5	2	3	3		3		7	5	8		3	2
1447928	1	71	6	5	4	1	1		1		7	5	8		3	2
1447954	2		6	5	3	1	2		1		7	5	8		5	3
1447961	2	67	6	5	2	2	2		3		7	5	8		3	2
1447971	1	61	6	5	2	2	4		3		3	1	1		3	2
1447972	1		6	5	3	3	2		4		7	5	8		2	2
1447972	2	64	6	5	2	2	3		3		7	5	8		3	2
1447997	2	65	6	5	2	2	3		3		7	5	8		1	1
1526526	1	73	6	5			1	3		9		5		1		1
1526547	2	67	6	5			3	4		9		5		1		1
1526561	2	69	6	5			1	1		9		5		1		1
1526593	2	70	6	5			1	4		9		5		1		1
1526595	2	76	6	5			1	1		9		5		1		1
1526612	1	81	6	5			3	3		9		5		1		1
1526620	1	63	6	5			3	2		4		2		1		1
1526646	2	79	6	5			1	1		9		5		1		1
1526668	1	64	6	5			2	1		9		5		1		1
1526695	2	62	6	5			2	4		9		5		1		1
1526708	2	70	6	5			2	4		9		5		1		1
1526739	2	83	6	5			1	1		9		5		1		1
1526741	1	63	6	5			2	2		4		2		1		1
1526751	2	77	6	5			1	4		4		2		1		1
1526765	1	75	6	5			1	4		9		5		1		1
1526766	2	66	6	5			3	4		9		5		1		1
1526777	1	60	6	5			2	4		9		5		1		1
1526778	2	74	6	5			1	3		9		5		1		1
1526787	1	64	6	5			2	4		9		5		1		1
1526788	2	61	6	5			3	4		9		5		1		1
1526802	1	67	6	5			2	3		9		5		1		1
1526814	1	81	6	5			2	3		9		5		1		1
1526820	1	63	6	5			2	3		9		5		1		1
1526844	2	66	6	5			1	1		9		5		1		1
1526851	2	70	6	5			1	3		9		5		1		1
1526915	1	67	6	5			2	2		7		4		1		1
1526918	2	79	6	5			1	4		9		5		1		1
1526925	2	82	6	5			1	3		9		5		1		1
1526926	1	86	6	5			2	4		9		5		1		1
1526936	2	72	6	5			1	1		9		5		1		1
1526937	1	77	6	5			2	1		9		5		1		1
1526952	2	66	6	5			2	4		9		5		2		2
1526972	2	80	6	5			2	3		9		5		2		2
1526976	1	65	6	5			2	3		9		5		2		2
1526982	2	61	6	5			3	4		9		5		2		2
1526997	1	71	6	5			1	1		9		5		2		2
1526998	2	61	6	5			3	1		9		5		2		2
1527009	2	62	6	5			4	2		3		1		2		2
1527019	2	64	6	5			2	3		9		5		2		2
1527043	2	65	6	5			3	3		3		1		2		2
1527056	2	78	6	5			3	4		9		5		2		2
1527072	1	73	6	5			1	3		9		5		2		2
1527083	2	60	6	5			2	1		9		5		2		2
1527094	2	68	6	5			4	4		9		5		2		2
1527095	1	68	6	5			1	2		9		5		2		2

1527096	2	70	6	5	1	1	9	5	2	2
1527111	2	60	6	5	3	2	3	1	2	2
1527157	2	64	6	5	2	3	9	5	2	2
1527169	1	61	6	5	2	1	4	2	2	2
1527173	2	60	6	5	1	2	9	5	2	2
1527176	2	69	6	5	1	4	9	5	2	2
1527186	2	60	6	5	2	2	7	4	2	2
1527211	2	63	6	5	2	4	9	5	3	2
1527218	2	60	6	5	1	2	9	5	3	2
1527258	2	63	6	5	2	2	9	5	3	2
1527264	2	60	6	5	3	1	4	2	3	2
1527314	2	70	6	5	2	3	9	5	3	2
1527327	2	85	6	5	3	1	9	5	3	2
1527348	2	60	6	5	2	1	9	5	3	2
1527366	2	70	6	5	3	3	9	5	3	2
1527367	1	62	6	5	2	4	4	2	3	2
1527368	2	60	6	5	4	3	1	1	3	2
1527377	1	64	6	5	2	3	9	5	3	2
1527378	2	69	6	5	3	3	4	2	3	2
1527394	2	71	6	5	3	3	9	5	3	2
1527399	1	85	6	5	3	3	9	5	3	2
1527413	2	79	6	5	1	1	9	5	3	2
1527431	1	60	6	5	2	3	9	5	3	2
1527434	2	65	6	5	3	1	9	5	3	2
1527439	2	61	6	5	1	1	7	4	3	2
1527458	2	69	6	5	2	3	9	5	3	2
1527509	2	68	6	5	3	3	9	5	4	3
1527514	2	67	6	5	2	3	9	5	4	3
1527515	1	62	6	5	4	3	9	5	4	3
1527516	2	60	6	5	3	3	2	1	4	3
1527523	1	61	6	5	2	2	9	5	4	3
1527524	2	63	6	5	3	4	9	5	4	3
1527546	1	77	6	5	4	3	9	5	4	3
1527551	2	79	6	5	3	1	9	5	4	3
1527572	2	87	6	5	4	1	9	5	4	3
1527578	2	65	6	5	3	2	9	5	4	3
1527966	2	62	6	5	2	2	9	5	1	1
1527968	1	70	6	5	1	1	9	5	1	1
1527996	2	71	6	5	1	1	9	5	1	1
1528012	1	77	6	5	2	3	9	5	1	1
1528014	2	63	6	5	3	3	9	5	1	1
1528240	2	81	6	5	2	1	9	5	1	1
1528246	2	67	6	5	3	3	9	5	3	2
1599026	2	65	6	5	2	4	9	5	1	1
1599028	1	65	6	5	3	4	2	1	1	1
1599058	1	60	6	5	2	4	9	5	1	1
1599076	2	65	6	5	2	2	9	5	1	1
1599080	1	68	6	5	2	3	9	5	1	1
1599085	2	60	6	5	3	3	4	2	1	1
1599091	2	88	6	5	1	1	9	5	1	1
1599126	2	78	6	5	1	4	9	5	1	1
1599127	1	60	6	5	2	2	4	2	1	1
1599130	2	75	6	5	1	4	9	5	1	1
1599146	2	71	6	5	3	4	9	5	1	1
1599149	1	72	6	5	1	4	9	5	1	1
1599163	2	73	6	5	2	2	9	5	1	1
1599167	1	75	6	5	2	3	9	5	1	1
1599168	2	72	6	5	1	1	9	5	1	1
1599188	1	61	6	5	2	1	9	5	1	1
1599190	2	64	6	5	1	1	9	5	1	1
1599194	2	73	6	5	1	3	9	5	1	1
1599196	2	61	6	5	2	3	6	3	1	1
1599215	2	74	6	5	2	1	9	5	1	1
1599226	2	67	6	5	2	2	9	5	1	1
1599229	1	70	6	5	2	3	9	5	1	1
1599248	2	61	6	5	3	3	9	5	1	1
1599251	2	62	6	5	2	3	9	5	1	1
1599253	1	65	6	5	3	3	4	2	1	1
1599274	1	65	6	5	3	3	9	5	1	1
1599294	1	64	6	5	1	3	6	3	1	1
1599299	2	73	6	5	1	2	9	5	1	1
1599318	2	66	6	5	3	3	9	5	1	1
1599327	1	70	6	5	2	1	9	5	1	1
1599331	2	66	6	5	2	1	9	5	1	1
1599340	2	65	6	5	2	3	9	5	1	1
1599344	1	76	6	5	2	2	9	5	1	1
1599350	2	70	6	5	3	2	9	5	1	1
1599407	1	62	6	5	2	2	9	5	1	1
1599408	2	62	6	5	2	2	4	2	1	1
1599422	1	62	6	5	2	2	9	5	1	1
1599451	2	63	6	5	3	3	9	5	2	2
1599478	1	62	6	5	3	3	9	5	2	2
1599484	2	63	6	5	3	4	1	1	2	2
1599509	1	72	6	5	2	4	9	5	2	2
1599510	2	75	6	5	2	1	9	5	2	2
1599512	1	60	6	5	2	3	4	2	2	2
1599539	1	73	6	5	2	2	9	5	2	2
1599584	1	62	6	5	1	1	9	5	2	2
1599615	1	67	6	5	2	3	9	5	2	2
1599638	2	61	6	5	3	1	9	5	2	2
1599639	1	64	6	5	1	4	3	1	2	2
1599659	2	67	6	5	2	1	9	5	2	2
1599661	1	73	6	5	2	1	9	5	2	2
1599662	2	69	6	5	1	3	9	5	2	2
1599670	2	63	6	5	2	3	9	5	2	2
1599671	1	67	6	5	1	2	9	5	2	2
1599699	1	81	6	5	2	3	9	5	2	2
1599713	2	72	6	5	2	2	9	5	3	2
1599721	1	71	6	5	1	2	9	5	3	2
1599757	1	60	6	5	2	2	9	5	3	2
1599760	2	67	6	5	1	1	9	5	3	2
1599773	2	80	6	5	2	2	9	5	3	2
1599785	2	76	6	5	3	2	9	5	3	2
1599811	2	63	6	5	1	2	9	5	3	2
1599814	1	68	6	5	2	4	9	5	3	2
1599829	2	62	6	5	2	3	9	5	3	2
1599838	1	61	6	5	2	3	4	2	3	2
1599839	2	74	6	5	2	1	4	2	3	2
1599843	2	62	6	5	1	1	10	7	3	2
1599844	1	60	6	5	1	1	10	7	3	2
1599862	1	69	6	5	4	3	9	5	3	2
1599879	1	62	6	5	1	3	9	5	3	2

1599880	2	62	6		5			3	3	2	1			3	2
1599881	2	68	6		5			3	2	9	5			3	2
1599909	1	75	6		5			2	2	9	5			3	2
1599910	2	61	6		5			4	3	9	5			3	2
1599915	2	68	6		5			2	1	9	5			3	2
1599918	1	61	6		5			4	4	3	1			3	2
1599922	2	61	6		5			4	4	1	1			3	2
1599942	1	60	6		5			3	4	1	1			3	2
1599950	1	63	6		5			2	3	9	5			3	2
1599955	2	64	6		5			2	2	10	7			3	2
1599959	1	60	6		5			3	1	9	5			3	2
1599972	1	80	6		5			2	4	9	5			4	3
1599991	1	63	6		5			3	2	9	5			4	3
1600005	2	60	6		5			4	4	9	5			4	3
1600011	2	66	6		5			3	2	9	5			4	3
1600016	2	60	6		5			3	4	4	2			4	3
1600017	1	61	6		5			3	3	9	5			4	3
1600018	2	60	6		5			3	3	5	2			4	3
1600025	1	67	6		5			4	3	9	5			4	3
1600035	2	78	6		5			1	1	9	5			4	3
1600057	2	67	6		5			2	4	4	2			4	3
1600058	1	65	6		5			2	2	9	5			4	3
1600061	2	80	6		5			3	1	9	5			4	3
1600074	2	62	6		5			4	4	9	5			4	3
1600082	2	65	6		5			4	2	9	5			4	3
1600084	1	65	6		5			2	2	9	5			4	3
1600095	2	71	6		5			2	1	9	5			4	3
1601211	2	72	6		5			1	3	9	5			1	1
1601254	1	68	6		5			2	3	9	5			1	1
1601346	2	63	6		5			2	3	9	5			2	2
1672929	2	63	6		5			1	3	9	5			1	1
1672944	1	67	6		5			3	2	9	5			1	1
1672946	2	80	6		5			2	4	9	5			1	1
1672954	1	69	6		5			2	1	9	5			1	1
1672961	1	60	6		5			2	3	9	5			1	1
1673007	1	69	6		5			3	3	9	5			1	1
1673019	2	60	6		5			2	4	9	5			1	1
1673034	2	69	6		5			2	3	9	5			1	1
1673044	2	86	6		5			2	1	9	5			1	1
1673045	1	82	6		5			2	1	9	5			1	1
1673059	2	69	6		5			3	3	9	5			1	1
1673132	1	69	6		5			1	3	9	5			1	1
1673133	2	63	6		5			2	3	9	5			1	1
1673159	2	70	6		5			1	3	9	5			1	1
1673160	1	63	6		5			2	4	9	5			1	1
1673161	2	71	6		5			1	3	9	5			1	1
1673220	1	76	6		5			1	1	9	5			1	1
1673224	2	70	6		5			2	1	9	5			1	1
1673264	1	76	6		5			2	4	9	5			1	1
1673270	2	63	6		5			1	2	9	5			1	1
1673271	1	70	6		5			2	2	9	5			1	1
1673289	2	63	6		5			2	3	9	5			1	1
1673295	1	67	6		5			2	3	9	5			1	1
1673296	2	60	6		5			2	3	7	4			1	1
1673309	1	63	6		5			3	3	1	1			1	1
1673313	2	69	6		5			3	3	9	5			1	1
1673340	1	70	6		5			2	1	9	5			2	2
1673349	2	98	6		5			1	1	9	5			2	2
1673350	1	62	6		5			3	1	9	5			2	2
1673371	1	61	6		5			2	4	4	2			2	2
1673408	2	71	6		5			1	3	9	5			2	2
1673420	1	63	6		5			2	3	4	2			2	2
1673443	2	66	6		5			1	3	9	5			2	2
1673471	2	60	6		5			2	4	9	5			2	2
1673472	1	60	6		5			2	4	9	5			2	2
1673482	1	68	6		5			2	4	9	5			2	2
1673483	2	73	6		5			2	3	9	5			2	2
1673496	2	71	6		5			1	2	9	5			2	2
1673509	2	67	6		5			1	3	9	5			2	2
1673512	1	60	6		5			3	4	4	2			2	2
1673522	1	63	6		5			2	4	4	2			2	2
1673550	2	64	6		5			4	3	9	5			2	2
1673560	1	65	6		5			2	2	9	5			2	2
1673573	2	69	6		5			3	2	9	5			2	2
1673592	1	69	6		5			2	3	9	5			2	2
1673610	1	62	6		5			3	1	4	2			3	2
1673631	2	62	6		5			3	3	4	2			3	2
1673632	1	72	6		5			2	2	9	5			3	2
1673655	1	74	6		5			2	3	9	5			3	2
1673664	2	61	6		5			2	4	9	5			3	2
1673716	2	60	6		5			2	3	9	5			3	2
1673719	1	60	6		5			1	3	9	5			3	2
1673734	1	63	6		5			2	3	4	2			3	2
1673735	2	71	6		5			1	1	9	5			3	2
1673743	1	79	6		5			4	2	9	5			3	2
1673746	2	71	6		5			1	3	9	5			3	2
1673753	2	69	6		5			2	1	9	5			3	2
1673777	1	63	6		5			3	3	9	5			3	2
1673778	2	63	6		5			3	3	9	5			3	2
1673779	2	83	6		5			1	1	9	5			3	2
1673780	1	60	6		5			3	3	4	2			3	2
1673853	2	77	6		5			3	2	9	5			3	2
1673898	2	63	6		5			2	1	9	5			4	3
1673904	1	66	6		5			1	2	9	5			4	3
1673914	2	84	6		5			2	4	9	5			4	3
1673916	2	74	6		5			2	3	9	5			4	3
1673951	2	67	6		5			3	3	2	1			4	3
1673955	2	67	6		5			3	1	9	5			4	3
1673956	1	66	6		5			3	3	9	5			4	3
1673959	2	63	6		5			3	3	2	1			4	3
1673964	1	64	6		5			3	3	9	5			4	3
1673972	2	84	6		5			4	2	9	5			4	3
1673980	2	69	6		5			3	2	9	5			4	3
1673986	2	63	6		5			3	4	9	5			4	3
1674658	1	62	6		5			2	1	4	2			2	2
1674670	2	60	6		5			4	4	9	5			3	2
1674696	1	64	6		5			2	3	10	7			1	1
1674728	1	63	6		5			1	3	9	5			1	1
1	2			5	5	2	8	2	1	9	5	4	3	3	2
80	2			5	5	2	8	4	3	9	5	2	3	2	2
81	1			5	5	2	7	3	4	9	5	2	1	3	2

96	2		5	5		2		8	3	4		9		5	2	3		4		3
109	2		5	5		1		8	2	1		4		2	4	3		3		2
113	2		5	5		5		4	3	2		9		5	3	1		1		1
125	2		5	5		2		7	3	2		3		1	3	3		3		2
132	1		5	5		2		8	3	4		9		5	2	3		2		2
136	1		5	5		1		7	2	1		10		7	5	3		2		2
162	2		5	5		1		8	3	1		9		5	3	1		2		2
175	1		5	5		2		7	2	3		9		5	3	3		1		1
189	2		5	5		2		8	3	2		9		5	3	3		2		2
240	1		5	5		2		7	2	1		10		7	5	3		2		2
273	2		5	5		2		7	3	4		2		1	2	2		4		3
278	2		5	5		1		8	2	1		9		5	4	3		2		2
290	2		5	5		1		8	2	1		9		5	4	3		2		2
292	2		5	5		2		8	4	4		9		5	2	2		2		2
298	2		5	5		2		8	3	2		9		5	3	2		3		2
314	1		5	5		1		8	2	1		9		5	5	3		2		2
317	1		5	5		2		7	2	3		9		5	3	3		3		2
328	1		5	5		4		8	3	4		9		5	1	1		3		2
333	2		5	5		2		8	2	1		9		5	4	3		3		2
366	2		5	5		1		8	1	1		9		5	5	3		3		2
384	1		5	5		2		8	3	3		9		5	3	1		3		2
399	1		5	5		1		8	4	2		9		5	3	3		2		2
401	2		5	5		1		8	2	1		9		5	5	3		2		2
417	1		5	5		1		8	3	1		9		5	3	3		4		3
460	1		5	5		1		8	2	1		9		5	4	2		1		1
474	2		5	5		1		7	3	2		4		2	3	3		3		2
482	2		5	5		4		6	4	4		9		5	1	1		4		3
489	2		5	5		2		8	3	3		9		5	3	3		3		2
495	2		5	5		1		8	3	1		9		5	3	3		1		1
497	2		5	5		2		8	2	2		9		5	4	3		1		1
512	2		5	5		4		8	1	1		9		5	5	3		2		2
516	2		5	5		2		8	2	3		6		3	3	3		1		1
537	2		5	5		1		8	2	1		9		5	4	3		4		3
542	1		5	5		3		6	3	4		9		5	2	3		3		2
572	1		5	5		1		8	1	1		6		3	5	3		1		1
582	1		5	5		4		8	2	3		6		3	3	3		1		1
590	1		5	5		2		8	1	1		9		5	5	3		1		1
610	1		5	5		2		8	1	2		9		5	4	3		2		2
618	2		5	5		4		6	1	3		9		5	4	3		1		1
630	2		5	5		1		8	4	2		9		5	3	3		4		3
662	1		5	5		5		6	2	1		9		5	4	2		1		1
664	1		5	5		2		8	1	1		9		5	5	3		1		1
666	2		5	5		3		8	2	1		9		5	4	2		3		2
681	2		5	5		2		8	3	1		9		5	3	1		3		2
682	1		5	5		5		5	2	3		9		5	4	2		3		2
731	2		5	5		1		8	3	1		9		5	3	3		3		2
742	1		5	5		3		7	2	4		9		5	1	1		4		3
751	2		5	5		1		8	3	1		9		5	3	3		1		1
764	2		5	5		3		6	3	2		9		5	3	3		3		2
795	2		5	5		3		6	3	2		9		5	3	3		3		2
802	1		5	5		2		8	3	4		9		5	2	2		2		2
806	1		5	5		2		8	3	1		9		5	3	3		2		2
814	1		5	5		2		8	2	3		9		5	3	3		2		2
818	2		5	5		1		8	3	1		9		5	3	3		2		2
847	2		5	5		3		5	1	3		9		5	4	3		2		2
869	1		5	5		2		7	3	3		9		5	3	3		3		2
874	2		5	5		5		5	1	3		9		5	4	3		1		1
875	2		5	5		1		8	3	1		9		5	3	2		3		2
899	2		5	5		2		7	2	3		4		2	3	3		3		2
938	2		5	5		3		6	2	3		5		2	3	2		2		2
971	2		5	5		4		8	2	4		9		5	3	3		3		2
983	2		5	5		1		7	3	1		3		1	3	2		4		3
1002	1		5	5		1		8	3	2		9		5	3	3		3		2
1009	2		5	5		1		8	3	1		9		5	3	3		1		1
1043	2		5	5		2		8	2	3		9		5	3	3		3		2
1064	2		5	5		4		6	3	3		9		5	3	3		2		2
1082	1		5	5		2		7	2	2		5		2	4	3		1		1
1083	1		5	5		4		5	2	2		4		2	3	2		1		1
1107	1		5	5		2		8	1	1		9		5	5	3		1		1
1125	2		5	5		1		8	3	1		9		5	3	3		3		2
1142	1		5	5		4		5	1	3		9		5	4	1		3		2
1148	1		5	5		2		8	2	2		9		5	3	3		4		3
1177	2		5	5		2		7	4	4		1		1	2	2		2		2
1178	1		5	5		2		7	4	4		1		1	1	2		4		3
1188	1		5	5		1		8	4	4		9		5	1	3		4		3
1214	1		5	5		2		8	1	2		9		5	4	3		3		2
1226	1		5	5		1		8	4	1		9		5	3	3		2		2
1247	2		5	5		3		8	1	2		9		5	4	3		2		2
1258	2		5	5		2		8	4	3		9		5	2	1		3		2
1294	1		5	5		1		8	2	1		6		3	5	3		1		1
1299	1		5	5		2		8	3	3		9		5	3	3		3		2
1327	2		5	5		2		8	3	2		9		5	3	3		1		1
1375	1		5	5		1		8	3	1		6		3	4	3		1		1
1382	2		5	5		1		8	3	1		9		5	3	3		1		1
1385	2		5	5		1		8	1	1		9		5	5	3		1		1
1406	1		5	5		2		7	3	4		1		1	1	1		2		2
1416	1		5	5		5		4	1	3		9		5	4	3		2		2
1421	2		5	5		1		8	3	1		9		5	4	3		2		2
1422	2		5	5		1		8	3	1		9		5	3	3		2		2
1427	1		5	5		2		8	1	1		9		5	5	3		2		2
1443	1		5	5		1		8	1	1		6		3	5	3		1		1
1499	1		5	5		2		8	3	1		9		5	3	3		3		2
1510	1	6		5	2		2		3		4		3	1			8		2	2
1513	2		5	5		2		8	1	1		6		3	5	3		1		1
1513	2	6		5	4		1		1		1		7	5			8		3	2
1538	1		5	5		2		8	1	2		9		5	4	3		1		1
1556	2		5	5		1		8	3	1		9		5	3	3		3		2
1560	1		5	5		1		8	3	1		9		5	4	3		3		2
1570	1		5	5		1		8	2	1		9		5	4	3		4		3
1587	1		5	5		2		7	2	1		10		7	4	3		1		1
1595	2		5	5		2		8	2	1		9		5	4	3		3		2
1616	1		5	5		2		7	2	2		4		2	4	2		3		2
1625	2		5	5		1		8	2	1		9		5	4	3		3		2
1632	1		5	5		2		8	4	3		9		5	2	3		2		2
1675	2		5	5		4		5	3	4		9		5	2	3		1		1
1677	2		5	5		5		5	3	3		9		5	2	3		1		1
1685	2		5	5		2		8	4	2		9		5	3	3		1		1
1700	2		5	5		2		8	1	2		9		5	4	3		1		1
1712	1		5	5		2		6	3	4		9		5	2	3		2		2
1723	1		5	5		2		8	2	2		9		5	4	3		2		2
1732	2		5	5		1		8	3	1		9		5	3	3		3		2

1748	1			5	5		3		7	4	4		9		5	1	1		4		3
1798	1			5	5		1		8	2	2		9		5	3	3		3		2
1811	1			5	5		2		8	1	2		6		3	4	3		3		2
1823	2			5	5		4		4	3	3		9		5	3	3		3		2
1828	1			5	5		2		8	1	1		9		5	5	3		1		1
1830	1			5	5		1		8	2	1		9		5	4	3		1		1
1855	2			5	5		2		8	3	2		9		5	3	3		1		1
1443761	2		6		5	2		2		3		3		7	5			8		1	1
1443771	2	66	6		5	2		2		4		3		7	5			1		1	1
1443779	2	66	6		5	4		1		2		2		7	5			8		1	1
1443782	2		6		5	4		1		4		2		7	5			1		1	1
1443790	2		6		5	2		2		2		4		5	3			8		1	1
1443795	2		6		5	2		5		2		3		7	5			8		1	1
1443813	2		6		5	4		1		1		1		7	5			8		1	1
1443817	2	61	6		5	2		4		3		4		7	5			8		1	1
1443817	1		6		5	1		2		1		2		7	5			8		1	1
1443823	2		6		5	2		2		3		4		7	5			8		1	1
1443837	2		6		5	4		1		2		1		7	5			8		1	1
1443842	2	70	6		5	2		2		3		4		7	5			8		1	1
1443853	2		6		5	4		5		1		4		7	5			8		1	1
1443854	1		6		5	2		2		1		2		7	5			8		1	1
1443861	2		6		5	2		2		1		3		7	5			2		1	1
1443871	2		6		5	4		1		1		1		7	5			8		1	1
1443889	2		6		5	2		2		2		3		7	5			8		1	1
1443898	1	67	6		5	2		2		2		3		7	5			8		1	1
1443908	2	63	6		5	2		2		2		4		7	5			8		1	1
1443930	2	61	6		5	4		1		4		4		7	5			8		1	1
1443931	1	61	6		5	3		1		4		4		7	5			8		1	1
1443941	2		6		5	3		1		2		1		7	5			8		1	1
1443952	2		6		5	2		2		2		4		7	5			8		1	1
1443985	1		6		5	2		2		1		3		7	5			8		1	1
1443987	2		6		5	2		2		2		4		7	5			8		1	1
1443988	1		6		5	2		2		2		3		7	5			8		1	1
1443995	1	82	6		5	2		2		1		4		7	5			8		1	1
1443996	2	73	6		5	2		2		1		3		7	5			8		1	1
1443997	2		6		5	4		2		4		4		7	5			8		1	1
1443999	2		6		5	2		2		3		2		7	5			2		1	1
1444009	2	60	6		5	2		2		2		4		7	5			8		1	1
1444045	1		6		5	2		2		3		3		1	1			2		1	1
1444062	2		6		5	2		2		2		2		7	5			8		1	1
1444066	1		6		5	2		2		2		3		4	2			2		1	1
1444076	1		6		5	4		1		2		2		7	5			8		1	1
1444079	2	71	6		5	2		3		1		3		7	5			8		1	1
1444086	1	63	6		5	2		2		2		2		7	5			5		1	1
1444099	2		6		5	2		2		3		2		7	5			8		1	1
1444108	1		6		5	2		2		2		4		4	2			8		1	1
1444126	2		6		5	2		2		1		2		7	5			8		1	1
1444134	1		6		5	2		3		2		4		4	2			6		1	1
1444196	2		6		5	2		2		2		4		7	5			8		2	2
1444206	2	72	6		5	4		1		2		2		7	5			8		2	2
1444220	2		6		5	2		2		1		1		7	5			2		2	2
1444227	2	60	6		5	2		3		2		3		4	2			3		2	2
1444244	1	78	6		5	2		2		1		2		7	5			8		2	2
1444247	2	76	6		5	4		1		1		1		7	5			8		2	2
1444248	2		6		5	4		1		2		2		7	5			8		2	2
1444263	2		6		5	2		2		3		4		3	1			1		2	2
1444264	1		6		5	2		2		3		3		2	1			1		2	2
1444271	2	63	6		5	2		2		3		4		7	5			2		2	2
1444272	1	70	6		5	4		2		4		4		7	5			1		2	2
1444276	2		6		5	4		1		1		1		7	5			8		2	2
1444280	2		6		5	2		2		1		3		7	5			8		2	2
1444288	1		6		5	2		2		3		3		7	5			8		2	2
1444333	2		6		5	2		2		3		3		7	5			1		2	2
1444336	1	75	6		5	2		2		3		4		7	5			8		3	2
1444337	2		6		5	2		2		3		3		3	1			2		2	2
1444353	2		6		5	4		1		1		1		7	5			8		3	2
1444354	1		6		5	2		2		2		3		4	2			8		3	2
1444362	1	61	6		5	2		2		4		4		1	1			1		3	2
1444363	2		6		5	2		2		1		4		7	5			8		3	2
1444363	2	66	6		5	4		1		2		4		7	5			8		3	2
1444385	2		6		5	4		2		3		4		7	5			8		3	2
1444392	2		6		5	2		2		4		3		2	1			1		3	2
1444393	2	80	6		5	4		2		2		3		7	5			8		3	2
1444394	1		6		5	4		1		1		4		7	5			8		3	2
1444408	1		6		5	2		2		1		3		7	5			2		3	2
1444416	2		6		5	4		1		1		1		7	5			8		3	2
1444417	1		6		5	2		2		2		3		7	5			8		3	2
1444432	1		6		5	2		2		2		3		7	5			8		3	2
1444437	1		6		5	2		2		3		4		7	5			1		3	2
1444450	2	68	6		5	3		1		3		4		7	5			8		3	2
1444450	2		6		5	2		2		1		3		7	5			8		3	2
1444455	2	62	6		5	2		2		4		3		7	5			3		3	2
1444468	1		6		5	2		2		2		4		7	5			8		3	2
1444475	2		6		5	4		1		1		4		7	5			8		3	2
1444482	2	65	6		5	4		2		2		4		7	5			8		3	2
1444482	1		6		5	2		4		2		3		7	5			6		3	2
1444483	1	60	6		5	2		2		2		4		4	2			1		3	2
1444484	2		6		5	2		2		2		3		7	5			8		3	2
1444488	2		6		5	2		3		4		3		7	5			1		3	2
1444489	1		6		5	2		2		3		3		7	5			8		3	2
1444498	2		6		5	4		1		3		1		7	5			8		3	2
1444499	1		6		5	2		2		2		3		7	5			1		3	2
1444514	2		6		5	2		2		2		2		7	5			8		3	2
1444534	2	63	6		5	2		2		3		3		7	5			8		4	2
1444538	2	72	6		5	4		1		3		2		7	5			8		4	2
1444553	1		6		5	3		1		2		4		7	5			8		3	2
1444554	2		6		5	3		1		3		4		7	5			8		3	2
1444568	2		6		5	4		1		1		1		7	5			8		4	2
1444570	2	60	6		5	2		3		2		1		6	4			8		4	2
1444578	2	71	6		5	4		2		4		4		7	5			8		4	2
1444580	1		6		5	2		2		4		4		7	5			2		4	2
1444583	2		6		5	4		1		3		2		7	5			8		4	2
1444586	1		6		5	2		2		3		4		7	5			2		4	2
1444594	2		6		5	2		3		2		3		4	2			8		4	2
1444598	2		6		5	4		1		1		1		7	5			8		4	2
1444609	2		6		5	4		1		1		1		7	5			8		4	2
1444630	2		6		5	2		4		1		3		9	7			8		4	2
1444633	1		6		5	2		2		2		4		7	5			8		4	2
1444634	2		6		5	2		2		3		4		7	5			8		4	2
1444643	2	72	6		5	4		1		3		1		7	5			8		4	2
1444644	1		6		5	3		1		3		2		7	5			8		4	2
1444662	1		6		5	2		2		2		3		7	5			8		4	2

1444687	2		6	5	2	3	2		4		7	5	8		4	2
1444691	2	62	6	5	3	1	3		2		4	2	8		4	2
1444710	2		6	5	4	1	1		3		7	5	8		4	2
1444719	2	61	6	5	2	2	3		4		4	2	1		5	3
1444720	1	67	6	5	2	2	3		4		9	7	8		5	3
1444721	1		6	5	2	2	1		3		7	5	8		4	2
1444722	2		6	5	2	2	3		4		4	2	99		4	2
1444727	1		6	5	4	1	2		4		9	7	8		4	2
1444739	2	66	6	5	4	2	1		4		7	5	8		5	3
1444765	1		6	5	4	1	1		2		7	5	8		5	3
1444769	2		6	5	4	1	1		1		7	5	8		5	3
1444775	2	71	6	5	4	1	3		1		7	5	8		5	3
1444775	1		6	5	2	3	2		4		3	1	1		5	3
1444786	2	61	6	5	2	2	3		4		7	5	8		5	3
1444790	2		6	5	4	2	4		3		7	5	8		5	3
1444795	2		6	5	2	3	3		4		7	5	8		5	3
1444796	1		6	5	2	2	3		4		1	1	2		5	3
1444803	2	68	6	5	2	2	4		3		7	5	1		5	3
1444804	1		6	5	2	2	2		2		7	5	8		5	3
1444804	1	67	6	5	2	2	4		3		7	5	1		5	3
1444806	2	71	6	5	2	2	3		3		7	5	1		5	3
1444807	2		6	5	4	3	4		4		7	5	2		5	3
1444809	2		6	5	2	2	3		4		7	5	2		5	3
1444815	1	68	6	5	2	2	3		3		7	5	8		5	3
1444816	2	61	6	5	4	1	4		3		3	1	1		5	3
1446170	1		6	5	2	3	3		3		7	5	99		5	3
1446186	2		6	5	4	1	3		2		7	5	8		5	3
1447508	2		6	5	2	2	3		3		7	5	1		5	3
1447533	1	69	6	5	4	3	1		4		7	5	99		1	1
1447533	1		6	5	2	5	3		3		4	2	8		5	3
1447535	2		6	5	4	1	3		2		7	5	2		5	3
1447561	2		6	5	2	2	2		3		7	5	1		5	3
1447580	2		6	5	4	2	1		4		4	2	8		5	3
1447583	1	60	6	5	2	2	4		4		7	5	1		1	1
1447583	2		6	5	4	1	1		1		7	5	8		1	1
1447923	2		6	5	4	1	1		1		7	5	8		5	3
1447952	1		6	5	3	2	2		3		4	2	8		4	2
1447952	1	60	6	5	2	2	2		2		7	5	8		1	1
1447967	2		6	5	2	2	3		3		7	5	8		1	1
1447967	2	67	6	5	4	1	3		1		7	5	8		2	2
1448008	1		6	5	2	3	2		4		7	5	8		4	2
1448009	2		6	5	2	1	3		1		7	5	8		1	1
1526594	2	60	6	5			2	2		6		3		1		1
1526625	1	76	6	5			1	2		9		5		1		1
1526637	1	72	6	5			1	3		9		5		1		1
1526638	2	67	6	5			2	3		9		5		1		1
1526643	1	63	6	5			2	1		9		5		1		1
1526702	2	64	6	5			2	1		9		5		1		1
1526732	1	67	6	5			2	4		9		5		1		1
1526764	2	72	6	5			2	4		9		5		1		1
1526797	2	78	6	5			1	4		9		5		1		1
1526811	2	66	6	5			1	1		9		5		1		1
1526884	2	72	6	5			2	2		9		5		1		1
1526888	1	77	6	5			1	3		9		5		1		1
1526900	2	72	6	5			2	3		9		5		1		1
1526939	2	62	6	5			3	1		3		1		2		2
1526950	1	60	6	5			3	4		3		1		2		2
1526971	1	82	6	5			1	3		9		5		2		2
1526995	2	61	6	5			1	1		9		5		2		2
1527010	1	61	6	5			4	2		3		1		2		2
1527032	1	60	6	5			1	2		10		7		2		2
1527033	2	74	6	5			1	1		9		5		2		2
1527104	2	67	6	5			3	4		9		5		2		2
1527146	2	77	6	5			3	4		9		5		2		2
1527187	1	60	6	5			1	1		9		5		2		2
1527188	2	60	6	5			1	1		9		5		2		2
1527219	1	66	6	5			2	3		9		5		3		2
1527250	2	60	6	5			1	1		9		5		3		2
1527255	1	70	6	5			3	3		3		1		3		2
1527272	1	62	6	5			2	3		9		5		3		2
1527273	2	63	6	5			3	3		9		5		3		2
1527309	2	67	6	5			2	3		9		5		3		2
1527312	1	60	6	5			4	4		1		1		3		2
1527321	2	68	6	5			1	1		9		5		3		2
1527325	2	82	6	5			3	1		9		5		3		2
1527336	1	63	6	5			2	2		9		5		3		2
1527337	2	74	6	5			1	2		9		5		3		2
1527353	2	66	6	5			3	3		9		5		3		2
1527421	1	66	6	5			3	4		1		1		3		2
1527456	2	61	6	5			2	3		9		5		3		2
1527457	1	62	6	5			2	3		7		4		3		2
1527469	2	66	6	5			2	3		9		5		4		3
1527484	2	74	6	5			3	1		9		5		4		3
1527503	2	73	6	5			3	2		9		5		4		3
1527542	1	61	6	5			2	3		3		1		4		3
1527555	2	69	6	5			3	3		9		5		4		3
1527593	2	79	6	5			3	4		9		5		4		3
1599033	2	72	6	5			1	2		9		5		1		1
1599045	1	66	6	5			2	2		9		5		1		1
1599056	2	63	6	5			1	3		9		5		1		1
1599092	2	70	6	5			1	1		9		5		1		1
1599094	1	67	6	5			2	1		9		5		1		1
1599257	1	65	6	5			2	2		9		5		1		1
1599258	2	70	6	5			1	2		9		5		1		1
1599275	2	85	6	5			1	2		9		5		1		1
1599290	2	71	6	5			1	2		9		5		1		1
1599321	2	62	6	5			3	4		9		5		1		1
1599334	1	62	6	5			2	2		4		2		1		1
1599356	2	67	6	5			2	3		9		5		1		1
1599380	1	70	6	5			1	1		9		5		1		1
1599427	2	61	6	5			3	2		10		7		1		1
1599454	2	61	6	5			2	2		9		5		2		2
1599465	2	61	6	5			3	2		9		5		2		2
1599499	1	82	6	5			4	3		9		5		2		2
1599534	1	60	6	5			3	4		4		2		2		2
1599538	2	60	6	5			2	2		4		2		2		2
1599557	1	70	6	5			3	3		9		5		2		2
1599591	2	62	6	5			2	3		9		5		2		2
1599592	1	67	6	5			2	3		4		2		2		2
1599625	1	60	6	5			2	1		9		5		2		2
1599627	2	70	6	5			2	2		9		5		2		2
1599700	2	63	6	5			3	4		9		5		2		2

1599712	1	60	6		5			2	2	4	2			3	2
1599746	1	67	6		5			2	2	4	2			3	2
1599761	1	60	6		5			2	1	9	5			3	2
1599816	2	69	6		5			2	3	9	5			3	2
1599852	1	60	6		5			3	1	3	1			3	2
1599863	2	65	6		5			2	3	9	5			3	2
1599901	1	63	6		5			4	3	1	1			3	2
1599930	2	67	6		5			2	2	9	5			3	2
1599971	2	75	6		5			3	1	9	5			4	3
1599983	2	61	6		5			1	2	9	5			4	3
1600006	1	64	6		5			2	4	4	2			4	3
1600033	2	81	6		5			1	1	9	5			4	3
1600053	2	60	6		5			2	2	4	2			4	3
1600088	2	62	6		5			2	4	9	5			4	3
1601258	2	74	6		5			4	4	9	5			4	3
1601803	1	77	6		5			3	2	9	5			2	2
1601862	1	61	6		5			3	2	9	5			2	2
1601916	2	79	6		5			2	3	9	5			1	1
1601921	1	67	6		5			2	2	9	5			4	3
1673020	1	85	6		5			1	3	9	5			1	1
1673032	1	72	6		5			1	2	9	5			1	1
1673033	2	68	6		5			1	2	9	5			1	1
1673137	2	67	6		5			1	1	9	5			1	1
1673183	2	69	6		5			1	1	9	5			1	1
1673196	1	78	6		5			2	3	9	5			1	1
1673207	1	61	6		5			3	2	4	2			1	1
1673323	2	80	6		5			2	3	9	5			1	1
1673352	2	64	6		5			4	2	2	1			2	2
1673365	2	67	6		5			2	3	9	5			2	2
1673372	2	61	6		5			2	3	9	5			2	2
1673385	1	73	6		5			2	2	9	5			2	2
1673407	1	68	6		5			2	3	9	5			2	2
1673410	1	62	6		5			2	3	9	5			2	2
1673419	2	61	6		5			3	3	3	1			2	2
1673421	2	68	6		5			2	3	9	5			2	2
1673444	2	62	6		5			4	4	9	5			2	2
1673445	1	74	6		5			3	4	9	5			2	2
1673460	1	62	6		5			2	1	9	5			2	2
1673494	2	63	6		5			1	1	9	5			2	2
1673553	1	63	6		5			3	4	1	1			2	2
1673609	2	67	6		5			4	4	9	5			3	2
1673611	2	76	6		5			1	1	9	5			3	2
1673617	1	60	6		5			1	1	9	5			3	2
1673618	2	72	6		5			3	1	9	5			3	2
1673651	2	68	6		5			4	3	9	5			3	2
1673672	1	78	6		5			4	4	9	5			3	2
1673682	1	60	6		5			2	4	4	2			3	2
1673683	2	63	6		5			2	4	9	5			3	2
1673794	2	60	6		5			3	2	4	2			3	2
1673827	1	72	6		5			2	4	9	5			3	2
1673870	1	76	6		5			2	3	9	5			4	3
1673879	2	83	6		5			1	1	9	5			4	3
1673884	2	62	6		5			3	1	3	1			4	3
1673993	2	60	6		5			2	2	9	5			4	3
2	2			5	5	2	8	3	3	9	5	3	3	3	2
24	1			5	5	2	7	3	1	10	7	3	1	2	2
39	2			5	5	2	8	1	2	9	5	4	3	3	2
63	2			5	5	3	6	1	2	6	3	4	3	1	1
75	2			5	5	1	8	1	1	6	3	5	3	1	1
82	1			5	5	1	8	1	1	9	5	5	3	2	2
102	2			5	5	2	8	2	1	9	5	4	3	1	1
117	2			5	5	1	8	3	2	9	5	3	3	4	3
119	1			5	5	3	6	2	3	4	2	3	3	4	3
123	2			5	5	1	8	2	1	9	5	4	3	4	3
128	1			5	5	1	7	3	3	1	1	2	1	3	2
144	1			5	5	2	7	2	2	5	2	4	3	3	2
144	1			5	5	4	8	1	3	6	3	4	3	1	1
154	2			5	5	2	8	3	3	9	5	2	3	3	2
158	2			5	5	1	8	3	1	9	5	3	2	2	2
159	2			5	5	2	8	3	2	9	5	3	1	2	2
160	1			5	5	1	8	4	2	9	5	3	2	2	2
188	2			5	5	1	8	2	1	9	5	5	3	2	2
204	2			5	5	1	8	4	2	9	5	3	3	3	2
218	1			5	5	2	8	2	3	9	5	3	3	4	3
228	2			5	5	1	8	3	1	9	5	3	2	2	2
281	1			5	5	2	7	2	2	4	2	4	3	2	2
282	1			5	5	2	8	2	3	9	5	3	1	2	2
283	1			5	5	2	8	4	4	9	5	1	2	3	2
285	1			5	5	2	8	3	2	9	5	3	2	3	2
286	1			5	5	2	5	4	1	2	1	3	1	2	2
299	2			5	5	1	8	1	1	9	5	5	3	3	2
317	1			5	5	3	8	2	3	9	5	3	3	3	2
344	1			5	5	3	8	2	3	9	5	3	1	2	2
345	2			5	5	2	8	4	4	9	5	2	2	2	2
350	2			5	5	2	8	3	3	9	5	2	2	2	2
390	2			5	5	1	7	2	2	5	2	4	3	3	2
395	1			5	5	3	8	2	4	9	5	3	3	3	2
414	1			5	5	2	8	4	3	9	5	2	3	4	3
420	1			5	5	2	8	1	1	9	5	5	3	1	1
438	2			5	5	1	8	3	2	9	5	3	3	2	2
451	2			5	5	3	6	3	4	9	5	2	1	3	2
458	1			5	5	2	8	3	4	9	5	2	3	3	2
468	2			5	5	1	8	3	1	9	5	3	3	3	2
485	1			5	5	2	8	3	2	9	5	3	1	3	2
510	1			5	5	3	8	2	2	9	5	4	3	1	1
513	1			5	5	1	8	2	1	9	5	5	3	2	2
536	1			5	5	1	8	3	1	9	5	3	2	4	3
561	2			5	5	1	8	3	1	9	5	3	3	2	2
574	2			5	5	1	8	1	1	9	5	5	3	1	1
583	1			5	5	1	8	2	1	9	5	5	3	3	2
585	2			5	5	1	8	3	1	9	5	3	1	3	2
586	2			5	5	1	8	1	1	9	5	5	3	1	1
601	2			5	5	1	8	1	1	9	5	5	3	3	2
640	1			5	5	2	7	2	2	9	5	4	3	1	1
673	2			5	5	2	8	3	1	9	5	3	3	1	1
676	2			5	5	2	8	2	1	6	3	5	3	1	1
683	2			5	5	5	6	2	1	5	2	4	3	2	2
720	1			5	5	2	7	2	1	3	1	4	3	3	2
723	2			5	5	1	8	1	1	9	5	5	3	3	2
727	1			5	5	2	8	3	1	9	5	3	2	2	2
737	1			5	5	1	7	4	2	4	2	3	3	4	3

739	1			5	5		2		7	3	4		4		2	1	2		4		3
745	1			5	5		3		8	2	4		9		5	2	2		1		1
749	2			5	5		2		8	3	1		9		5	4	3		1		1
768	2			5	5		1		7	3	3		2		1	3	2		2		2
796	1			5	5		2		8	2	3		9		5	3	3		2		2
799	1			5	5		1		8	3	2		9		5	3	2		2		2
811	1			5	5		2		8	2	4		9		5	3	3		3		2
816	1			5	5		1		8	3	2		9		5	2	2		2		2
819	1			5	5		2		8	3	4		9		5	2	1		3		2
829	1			5	5		1		8	2	1		9		5	4	3		3		2
833	1			5	5		2		7	2	2		9		5	4	3		4		3
835	2			5	5		3		7	2	4		4		2	1	2		4		3
836	2			5	5		2		7	2	1		4		2	5	2		4		3
838	2			5	5		3		6	2	3		5		2	3	2		3		2
839	2			5	5		2		6	3	1		3		1	3	2		3		2
841	2			5	5		2		6	4	4		9		5	2	3		2		2
842	1			5	5		2		8	4	4		9		5	2	1		2		2
857	2			5	5		5		6	1	4		6		3	2	3		1		1
876	1			5	5		2		8	2	2		9		5	4	3		1		1
884	1			5	5		2		7	2	3		4		2	3	3		2		2
891	2			5	5		1		8	1	1		6		3	5	3		1		1
899	1			5	5		4		6	2	2		9		5	4	3		1		1
900	2			5	5		2		8	1	2		9		5	4	3		1		1
903	2			5	5		2		8	2	1		6		3	5	3		1		1
932	1			5	5		3		6	4	4		9		5	1	2		2		2
939	2			5	5		2		7	2	2		4		2	4	3		2		2
940	1			5	5		4		5	3	2		10		7	3	1		3		2
943	1			5	5		1		7	2	1		4		2	4	3		3		2
945	2			5	5		2		8	4	4		9		5	1	3		3		2
948	2			5	5		3		6	4	4		9		5	1	1		3		2
1005	1			5	5		1		8	2	1		9		5	4	3		2		2
1006	2			5	5		4		4	2	4		9		5	3	2		4		3
1012	1			5	5		4		5	3	4		3		1	1	3		1		1
1015	2			5	5		1		8	2	2		9		5	4	3		4		3
1017	1			5	5		1		8	2	1		9		5	4	3		3		2
1024	1			5	5		2		8	1	2		9		5	5	3		1		1
1030	2			5	5		4		5	1	3		6		3	4	3		1		1
1036	2			5	5		2		8	3	4		9		5	2	3		4		3
1074	2			5	5		2		8	4	3		9		5	2	1		3		2
1079	2			5	5		3		8	3	3		9		5	2	2		1		1
1081	2			5	5		4		6	2	2		9		5	4	3		1		1
1099	1			5	5		3		8	2	1		9		5	4	3		2		2
1106	2			5	5		2		8	1	2		9		5	4	3		2		2
1109	1			5	5		1		8	1	1		5		2	5	3		2		2
1130	2			5	5		1		8	2	1		9		5	5	3		1		1
1149	1			5	5		4		7	2	4		4		2	2	1		4		3
1159	1			5	5		2		7	3	3		3		1	2	3		4		3
1164	2			5	5		1		8	3	1		9		5	3	2		4		3
1172	1			5	5		2		8	3	2		9		5	3	3		2		2
1184	2			5	5		1		8	4	1		9		5	3	2		4		3
1186	2			5	5		2		8	4	3		9		5	2	2		4		3
1188	2			5	5		2		8	3	3		9		5	2	1		4		3
1198	2			5	5		2		8	3	2		9		5	3	3		3		2
1251	2			5	5		2		8	3	2		9		5	3	2		3		2
1264	2			5	5		1		8	2	1		6		3	5	3		1		1
1300	2			5	5		2		8	3	3		9		5	2	2		3		2
1312	2			5	5		2		7	4	3		2		1	2	1		4		3
1340	1			5	5		1		8	2	1		9		5	5	3		1		1
1342	1			5	5		2		8	2	2		9		5	3	3		1		1
1345	1			5	5		1		8	2	1		9		5	5	3		1		1
1358	1			5	5		1		7	3	1		10		7	4	3		1		1
1384	1			5	5		2		8	2	2		9		5	4	3		1		1
1405	2			5	5		1		8	2	1		9		5	4	3		2		2
1408	2			5	5		1		8	3	1		9		5	3	3		3		2
1413	2			5	5		2		8	1	2		9		5	4	3		2		2
1414	2			5	5		1		8	1	1		9		5	5	3		3		2
1434	1			5	5		5		5	1	3		6		3	3	3		1		1
1470	2			5	5		1		8	2	1		9		5	4	3		1		1
1475	1			5	5		2		8	4	2		9		5	2	2		1		1
1480	1			5	5		2		8	2	3		9		5	3	3		2		2
1492	1			5	5		2		8	3	2		9		5	3	1		3		2
1510	2			5	5		1		8	1	1		9		5	5	3		3		2
1531	2			5	5		2		8	1	1		9		5	5	1		1		1
1546	2			5	5		1		8	2	1		9		5	4	3		2		2
1550	2			5	5		1		7	3	1		3		1	3	1		3		2
1562	2			5	5		1		8	3	1		9		5	3	3		3		2
1585	2			5	5		1		8	1	1		9		5	5	3		1		1
1613	1			5	5		2		8	2	1		9		5	5	3		3		2
1629	1			5	5		3		8	1	4		9		5	3	3		1		1
1643	1			5	5		4		6	2	2		9		5	4	3		1		1
1646	1			5	5		2		8	2	3		9		5	3	3		2		2
1648	1			5	5		3		8	2	4		9		5	3	3		1		1
1660	1			5	5		2		8	2	2		9		5	4	3		2		2
1673	2			5	5		1		8	3	1		4		2	3	3		3		2
1674	1			5	5		2		8	1	2		9		5	5	3		3		2
1694	1			5	5		2		7	4	4		2		1	1	1		3		2
1697	2			5	5		1		8	3	2		9		5	3	2		3		2
1702	1			5	5		1		8	1	1		9		5	5	3		1		1
1712	1			5	5		2		8	2	3		6		3	3	3		2		2
1736	2			5	5		2		8	3	4		9		5	2	1		4		3
1749	2			5	5		1		8	3	1		9		5	3	2		4		3
1758	2			5	5		3		8	3	4		9		5	1	2		4		3
1759	2			5	5		5		6	3	4		9		5	2	3		4		3
1760	1			5	5		2		7	3	2		4		2	3	3		4		3
1763	1			5	5		2		8	3	2		9		5	3	1		4		3
1768	1			5	5		2		8	1	2		9		5	4	3		2		2
1770	2			5	5		2		7	3	3		9		5	3	1		3		2
1776	2			5	5		1		8	3	2		9		5	3	3		3		2
1777	2			5	5		2		8	2	2		9		5	4	3		1		1
1778	2			5	5		1		8	1	1		9		5	5	3		1		1
1789	1			5	5		2		8	1	2		9		5	4	3		1		1
1809	1			5	5		1		8	3	1		9		5	3	2		3		2
1813	2			5	5		2		7	2	3		9		5	3	2		3		2
1819	2			5	5		2		8	3	2		9		5	3	3		3		2
1842	1			5	5		3		8	3	4		9		5	2	2		4		3
1865	1			5	5		2		8	1	2		9		5	4	3		3		2
1870	2			5	5		2		8	1	2		9		5	4	3		3		2
1872	1			5	5		1		8	2	1		9		5	5	3		3		2
1873	1			5	5		2		8	2	2		9		5	4	3		3		2
1443757	1	74	6		5	2		2		3		3		4	2			8		1	1
1443758	1		6		5	2		2		3		3		7	5			8		1	1

1443762	2		6	5	2	2	1	4	7	5	8	1	1
1443766	1	60	6	5	4	2	1	4	5	3	8	1	1
1443777	1		6	5	2	2	2	2	7	5	8	1	1
1443792	1	68	6	5	2	2	2	3	7	5	8	1	1
1443816	1	62	6	5	3	2	2	2	7	5	8	1	1
1443820	1	61	6	5	2	2	1	4	7	5	8	1	1
1443826	2	81	6	5	4	2	3	1	7	5	8	1	1
1443830	2	64	6	5	2	2	3	4	7	5	1	1	1
1443832	2		6	5	2	2	2	2	5	3	8	1	1
1443841	1		6	5	2	2	2	4	7	5	8	1	1
1443861	2	65	6	5	2	3	2	4	7	5	8	1	1
1443862	1	66	6	5	2	2	2	3	7	5	8	1	1
1443867	2	61	6	5	3	1	2	1	7	5	8	1	1
1443870	1		6	5	4	1	2	2	5	3	8	1	1
1443872	2		6	5	2	4	1	4	7	5	8	1	1
1443891	1	70	6	5	2	3	2	3	7	5	8	1	1
1443907	2		6	5	2	1	3	2	3	1	2	1	1
1443915	2	61	6	5	2	2	2	3	7	5	8	1	1
1443916	1	61	6	5	4	1	1	2	4	2	8	1	1
1443916	1		6	5	2	5	2	4	7	5	8	1	1
1443918	2		6	5	4	1	1	1	7	5	8	1	1
1443924	2		6	5	4	4	2	4	7	5	8	1	1
1443925	1		6	5	2	2	1	4	7	5	8	1	1
1443936	2		6	5	3	1	2	2	5	3	8	1	1
1443939	1		6	5	2	2	3	3	4	2	1	1	1
1443943	1		6	5	4	3	2	4	7	5	8	1	1
1443946	2	62	6	5	4	2	1	2	7	5	8	1	1
1443959	2		6	5	2	2	1	4	7	5	8	1	1
1443959	1	64	6	5	2	2	1	2	7	5	8	1	1
1443960	1		6	5	2	2	2	4	7	5	8	1	1
1443972	2		6	5	2	2	2	2	7	5	8	1	1
1443982	2	63	6	5	2	2	2	3	7	5	8	1	1
1443983	1	65	6	5	2	2	1	3	7	5	8	1	1
1443984	2	63	6	5	2	2	2	3	4	2	8	1	1
1443984	2		6	5	4	1	1	1	7	5	8	1	1
1443998	1		6	5	4	3	2	4	7	5	8	1	1
1444029	2	69	6	5	2	2	1	4	7	5	8	1	1
1444037	1		6	5	2	3	2	3	4	2	8	1	1
1444052	1		6	5	2	2	3	3	7	5	8	1	1
1444052	1	64	6	5	2	4	1	4	7	5	8	1	1
1444062	2	75	6	5	4	1	1	2	7	5	3	1	1
1444072	2	60	6	5	4	3	1	4	7	5	8	1	1
1444077	1	72	6	5	1	1	3	1	7	5	8	1	1
1444081	2		6	5	2	2	2	2	6	4	8	1	1
1444082	2		6	5	4	1	4	2	7	5	8	1	1
1444113	2		6	5	2	2	2	4	4	2	8	1	1
1444118	2	77	6	5	4	1	2	2	7	5	8	1	1
1444121	2		6	5	4	1	1	1	7	5	8	1	1
1444125	2	72	6	5	4	1	2	4	7	5	8	1	1
1444133	1	74	6	5	2	2	2	4	7	5	8	1	1
1444134	2	71	6	5	4	1	1	4	7	5	8	1	1
1444143	1		6	5	2	2	1	4	7	5	8	1	1
1444143	1	65	6	5	2	2	2	4	7	5	8	1	1
1444146	2	72	6	5	4	1	3	2	7	5	8	1	1
1444154	2	70	6	5	4	1	1	1	7	5	8	1	1
1444163	1	70	6	5	2	2	2	3	7	5	2	2	2
1444173	1	62	6	5	2	2	2	4	4	2	8	2	2
1444194	1	81	6	5	2	2	1	3	7	5	8	2	2
1444197	1		6	5	2	2	3	3	7	5	1	2	2
1444199	2		6	5	4	1	2	1	7	5	8	2	2
1444199	1	66	6	5	2	2	2	3	7	5	2	2	2
1444209	1		6	5	2	2	1	4	7	5	8	2	2
1444210	2		6	5	2	2	2	4	7	5	8	2	2
1444213	2		6	5	4	4	4	3	7	5	1	2	2
1444222	2		6	5	4	2	3	4	7	5	8	2	2
1444223	1		6	5	2	2	4	4	7	5	1	2	2
1444224	2	60	6	5	2	3	1	3	7	5	8	2	2
1444226	1	65	6	5	2	2	2	4	7	5	8	2	2
1444233	2		6	5	2	2	1	3	6	4	8	2	2
1444243	2	63	6	5	2	3	2	4	7	5	2	2	2
1444247	1		6	5	3	2	2	4	7	5	2	2	2
1444249	2		6	5	3	1	3	4	1	1	8	2	2
1444250	1		6	5	3	1	2	4	1	1	8	2	2
1444256	2	61	6	5	4	1	3	2	3	1	3	2	2
1444257	1	68	6	5	2	2	3	3	7	5	1	2	2
1444267	2		6	5	2	2	2	4	7	5	8	2	2
1444288	1	60	6	5	2	3	2	4	1	1	8	2	2
1444301	2	83	6	5	1	1	4	2	7	5	8	2	2
1444335	2	67	6	5	4	1	2	4	7	5	8	3	2
1444351	2	65	6	5	4	1	2	1	4	2	8	3	2
1444352	2		6	5	4	1	3	1	7	5	8	2	2
1444364	1		6	5	4	1	2	2	7	5	8	3	2
1444373	2		6	5	3	1	2	2	7	5	8	3	2
1444381	1	69	6	5	3	1	3	2	7	5	2	3	2
1444382	2		6	5	4	1	1	4	7	5	8	3	2
1444403	2		6	5	2	2	1	2	7	5	8	3	2
1444410	1	68	6	5	3	1	2	1	7	5	8	3	2
1444422	2		6	5	4	1	1	1	7	5	8	3	2
1444422	2	72	6	5	2	2	2	2	7	5	8	3	2
1444436	2		6	5	2	3	2	4	4	2	2	3	2
1444440	2	74	6	5	4	1	3	1	7	5	8	3	2
1444441	1	79	6	5	3	1	2	2	7	5	8	3	2
1444451	1	76	6	5	2	2	2	4	7	5	8	3	2
1444462	2	68	6	5	1	2	4	4	3	1	1	3	2
1444464	2		6	5	2	2	3	3	7	5	8	3	2
1444466	2	75	6	5	2	2	4	4	7	5	8	3	2
1444474	1		6	5	4	1	1	4	7	5	1	3	2
1444481	2		6	5	2	2	2	3	7	5	8	3	2
1444517	2	65	6	5	4	1	2	4	7	5	8	4	2
1444518	1	67	6	5	3	2	2	4	7	5	8	4	2
1444521	2		6	5	4	1	2	2	7	5	8	3	2
1444537	1	68	6	5	2	3	3	4	7	5	8	4	2
1444545	2	62	6	5	4	1	3	1	4	2	1	4	2
1444549	2	64	6	5	2	2	2	3	3	1	1	4	2
1444550	1	60	6	5	2	2	2	4	7	5	8	4	2
1444559	2	60	6	5	2	2	2	3	3	1	1	4	2
1444571	1	60	6	5	2	2	2	3	7	5	1	4	2
1444589	2	60	6	5	2	2	3	4	7	5	8	4	2
1444590	1	66	6	5	2	2	3	3	7	5	8	4	2
1444591	2	63	6	5	3	2	4	4	7	5	1	4	2
1444595	1		6	5	2	2	2	3	3	1	2	4	2
1444601	2	77	6	5	4	1	4	3	7	5	2	4	2

1444602	2	71	6	5	4	1	3		2		7	5	8		4	2
1444603	1	70	6	5	4	3	2		4		7	5	8		4	2
1444622	2	62	6	5	3	1	3		1		7	5	8		4	2
1444650	2	67	6	5	3	1	3		1		7	5	8		4	2
1444651	1	75	6	5	4	1	3		2		7	5	8		4	2
1444654	1		6	5	2	2	2		2		7	5	8		4	2
1444654	2	61	6	5	2	2	3		3		7	5	8		4	2
1444655	2		6	5	2	2	1		2		7	5	8		4	2
1444667	1	81	6	5	2	2	4		4		7	5	5		4	2
1444671	2	63	6	5	3	2	3		4		7	5	4		4	2
1444680	2		6	5	1	5	3		4		7	5	8		4	2
1444689	2		6	5	4	1	2		2		7	5	8		4	2
1444692	2	68	6	5	2	2	4		4		7	5	1		5	3
1444702	1		6	5	2	2	1		4		7	5	8		4	2
1444703	2		6	5	4	1	3		3		7	5	8		4	2
1444703	1	69	6	5	2	2	1		2		7	5	8		5	3
1444711	1		6	5	2	3	2		3		7	5	8		4	2
1444712	1	61	6	5	4	1	3		4		4	2	1		5	3
1444721	2	60	6	5	2	2	2		4		9	7	8		5	3
1444724	2		6	5	1	1	4		2		3	1	8		4	2
1444727	1	60	6	5	2	2	3		4		3	1	2		5	3
1444731	2		6	5	4	2	1		4		7	5	8		4	2
1444740	2		6	5	4	2	1		4		7	5	8		4	2
1444746	1	69	6	5	2	2	3		4		7	5	1		5	3
1444755	1	60	6	5	2	4	3		3		3	1	1		5	3
1444756	2	68	6	5	4	1	3		2		7	5	1		5	3
1444764	2		6	5	2	2	3		4		7	5	8		5	3
1444765	1	67	6	5	4	5	2		4		7	5	8		5	3
1444766	2	63	6	5	4	4	2		4		7	5	8		5	3
1444778	1	66	6	5	4	1	3		3		1	1	1		5	3
1444784	2		6	5	3	1	2		1		7	5	8		5	3
1444789	2	73	6	5	4	1	2		1		7	5	8		5	3
1444795	1	71	6	5	2	2	3		3		7	5	2		5	3
1444797	2	63	6	5	2	2	4		3		1	1	1		5	3
1444808	1		6	5	2	2	4		4		7	5	2		5	3
1446184	2	63	6	5	2	3	1		4		7	5	8		2	2
1446189	1		6	5	2	2	3		3		7	5	1		5	3
1446189	2	66	6	5	4	1	2		1		7	5	8		1	1
1446190	2	60	6	5	4	1	2		2		7	5	8		1	1
1446620	1	60	6	5	2	2	4		4		1	1	1		5	3
1447051	2	61	6	5	4	2	4		3		3	1	1		1	1
1447532	2	63	6	5	4	1	2		4		4	2	8		1	1
1447541	2	60	6	5	4	1	1		2		7	5	8		1	1
1447562	1		6	5	2	2	4		3		3	1	8		5	3
1447564	2		6	5	4	1	2		1		7	5	8		5	3
1447579	1		6	5	2	2	3		4		3	1	8		5	3
1447921	2		6	5	4	1	1		1		7	5	8		5	3
1447922	1		6	5	2	2	3		3		4	2	8		5	3
1447945	1		6	5	2	2	4		4		2	1	8		4	2
1447954	1	71	6	5	2	2	2		4		7	5	8		2	2
1447960	2		6	5	4	1	3		4		7	5	8		4	2
1448004	2		6	5	1	1	4		4		1	1	8		2	2
1448006	1	64	6	5	2	2	2		3		7	5	8		1	1
1448009	2	70	6	5	4	1	1		1		7	5	8		1	1
1448014	1	71	6	5	1	1	2		1		7	5	8		2	2
1526524	2	75	6	5			2	4		9		5		1		1
1526539	1	67	6	5			3	3		9		5		1		1
1526541	2	61	6	5			3	1		9		5		1		1
1526548	1	62	6	5			3	4		3		1		1		1
1526557	2	67	6	5			4	3		9		5		1		1
1526558	1	63	6	5			1	1		10		7		1		1
1526559	1	64	6	5			1	1		9		5		1		1
1526564	2	62	6	5			2	3		9		5		1		1
1526566	1	66	6	5			2	1		9		5		1		1
1526588	1	77	6	5			1	1		9		5		1		1
1526597	1	71	6	5			3	3		9		5		1		1
1526603	2	88	6	5			1	1		9		5		1		1
1526607	2	62	6	5			1	4		9		5		1		1
1526619	2	63	6	5			2	1		9		5		1		1
1526623	2	82	6	5			1	1		9		5		1		1
1526624	2	78	6	5			1	2		9		5		1		1
1526639	2	62	6	5			2	1		9		5		1		1
1526655	1	65	6	5			1	2		9		5		1		1
1526656	2	68	6	5			2	2		9		5		1		1
1526671	2	68	6	5			3	3		9		5		1		1
1526681	1	61	6	5			2	3		4		2		1		1
1526683	2	77	6	5			2	1		9		5		1		1
1526692	2	67	6	5			1	1		9		5		1		1
1526693	1	61	6	5			3	3		9		5		1		1
1526703	1	60	6	5			2	4		6		3		1		1
1526714	2	63	6	5			2	2		9		5		1		1
1526716	1	62	6	5			3	4		9		5		1		1
1526729	2	60	6	5			2	3		4		2		1		1
1526750	1	83	6	5			1	1		9		5		1		1
1526754	2	67	6	5			3	4		9		5		1		1
1526755	1	77	6	5			2	1		9		5		1		1
1526792	1	70	6	5			1	1		9		5		1		1
1526806	2	63	6	5			2	4		9		5		1		1
1526833	2	62	6	5			4	3		9		5		1		1
1526837	1	61	6	5			2	4		10		7		1		1
1526843	2	62	6	5			2	1		9		5		1		1
1526849	1	79	6	5			2	2		9		5		1		1
1526858	1	70	6	5			3	3		9		5		1		1
1526859	2	71	6	5			2	3		9		5		1		1
1526868	2	73	6	5			2	2		9		5		1		1
1526871	2	62	6	5			1	1		9		5		1		1
1526873	1	61	6	5			1	1		4		2		1		1
1526904	1	61	6	5			2	1		6		3		1		1
1526920	1	73	6	5			1	2		9		5		1		1
1526921	2	69	6	5			1	1		9		5		1		1
1526940	1	63	6	5			4	3		3		1		2		2
1526949	2	80	6	5			1	1		9		5		2		2
1526962	1	61	6	5			3	1		10		7		2		2
1526963	2	68	6	5			2	1		9		5		2		2
1526965	2	71	6	5			3	3		9		5		2		2
1526984	2	66	6	5			3	4		9		5		2		2
1526985	1	70	6	5			2	3		9		5		2		2
1527007	1	69	6	5			2	1		9		5		2		2
1527008	2	72	6	5			2	1		9		5		2		2
1527020	1	78	6	5			2	2		9		5		2		2
1527021	2	71	6	5			2	4		9		5		2		2
1527036	2	64	6	5			3	3		9		5		2		2

1527037	1	66	6	5	2	2	9	5	2	2
1527039	2	66	6	5	2	2	9	5	2	2
1527040	1	66	6	5	2	3	4	2	2	2
1527055	1	72	6	5	3	3	9	5	2	2
1527059	2	60	6	5	3	3	3	1	2	2
1527060	1	72	6	5	3	3	9	5	2	2
1527071	2	70	6	5	1	1	9	5	2	2
1527073	2	67	6	5	2	4	9	5	2	2
1527082	1	67	6	5	1	1	9	5	2	2
1527084	2	68	6	5	3	2	9	5	2	2
1527085	1	71	6	5	2	4	10	7	2	2
1527108	1	61	6	5	3	3	3	1	2	2
1527113	1	60	6	5	1	2	9	5	2	2
1527114	2	71	6	5	3	3	9	5	2	2
1527122	2	72	6	5	1	3	9	5	2	2
1527123	1	73	6	5	2	2	9	5	2	2
1527125	2	62	6	5	3	3	9	5	2	2
1527132	1	60	6	5	2	3	4	2	2	2
1527133	2	62	6	5	1	1	9	5	2	2
1527136	2	80	6	5	3	4	9	5	2	2
1527137	1	78	6	5	2	4	9	5	2	2
1527149	2	73	6	5	1	3	9	5	2	2
1527150	1	83	6	5	1	1	9	5	2	2
1527159	1	62	6	5	3	1	9	5	2	2
1527160	2	63	6	5	2	3	9	5	2	2
1527168	2	75	6	5	2	1	9	5	2	2
1527174	1	70	6	5	2	3	9	5	2	2
1527197	1	65	6	5	4	2	4	2	2	2
1527198	2	67	6	5	3	1	9	5	2	2
1527199	2	60	6	5	2	1	9	5	3	2
1527200	1	67	6	5	2	2	9	5	3	2
1527209	2	63	6	5	2	4	9	5	3	2
1527210	1	75	6	5	2	3	9	5	3	2
1527224	2	70	6	5	2	3	9	5	3	2
1527227	1	72	6	5	2	3	9	5	3	2
1527232	2	68	6	5	4	4	9	5	3	2
1527235	2	63	6	5	2	1	9	5	3	2
1527238	1	66	6	5	2	2	9	5	3	2
1527239	2	80	6	5	1	3	9	5	3	2
1527244	1	61	6	5	2	3	9	5	3	2
1527247	2	64	6	5	4	4	9	5	3	2
1527259	1	62	6	5	1	1	9	5	3	2
1527271	2	62	6	5	2	3	9	5	3	2
1527277	1	70	6	5	3	3	9	5	3	2
1527278	2	77	6	5	1	3	9	5	3	2
1527284	2	76	6	5	1	1	9	5	3	2
1527285	1	61	6	5	2	4	4	2	3	2
1527294	2	77	6	5	1	1	9	5	3	2
1527295	1	69	6	5	2	1	9	5	3	2
1527296	2	78	6	5	1	3	9	5	3	2
1527301	2	70	6	5	1	1	9	5	3	2
1527302	1	60	6	5	2	2	4	2	3	2
1527320	1	67	6	5	1	1	9	5	3	2
1527326	1	60	6	5	2	1	9	5	3	2
1527341	2	78	6	5	2	3	9	5	3	2
1527342	1	69	6	5	2	3	9	5	3	2
1527350	1	62	6	5	2	3	9	5	3	2
1527360	1	64	6	5	3	4	4	2	3	2
1527361	2	83	6	5	3	4	9	5	3	2
1527379	2	65	6	5	1	3	9	5	3	2
1527380	1	64	6	5	2	4	9	5	3	2
1527389	2	61	6	5	3	2	9	5	3	2
1527390	1	71	6	5	3	1	9	5	3	2
1527391	2	63	6	5	3	1	9	5	3	2
1527404	2	60	6	5	3	2	4	2	3	2
1527416	1	60	6	5	3	4	4	2	3	2
1527419	2	67	6	5	3	4	9	5	3	2
1527420	2	68	6	5	1	1	9	5	3	2
1527428	2	62	6	5	3	2	3	1	3	2
1527440	1	71	6	5	2	3	9	5	3	2
1527443	2	63	6	5	4	1	9	5	3	2
1527448	1	88	6	5	4	4	9	5	3	2
1527453	2	83	6	5	1	4	7	4	3	2
1527467	1	68	6	5	3	4	9	5	3	2
1527468	2	62	6	5	3	3	9	5	3	2
1527470	1	62	6	5	2	4	6	3	4	3
1527479	2	72	6	5	1	1	9	5	4	3
1527480	1	67	6	5	2	2	9	5	4	3
1527481	2	69	6	5	3	3	9	5	4	3
1527489	1	62	6	5	1	4	4	2	4	3
1527496	2	65	6	5	3	4	9	5	4	3
1527497	1	70	6	5	3	4	9	5	4	3
1527498	2	69	6	5	3	4	9	5	4	3
1527504	1	61	6	5	4	3	4	2	4	3
1527543	2	62	6	5	3	2	9	5	4	3
1527544	2	61	6	5	3	4	3	1	4	3
1527556	1	67	6	5	3	4	3	1	4	3
1527559	2	60	6	5	3	4	3	1	4	3
1527564	1	77	6	5	2	2	9	5	4	3
1527565	2	63	6	5	4	4	3	1	4	3
1527574	1	62	6	5	2	4	1	1	4	3
1527580	2	81	6	5	3	4	9	5	4	3
1527582	1	64	6	5	4	4	1	1	4	3
1527586	2	62	6	5	1	3	4	2	4	3
1527592	1	80	6	5	3	3	9	5	4	3
1527979	1	64	6	5	2	3	9	5	4	3
1527980	2	61	6	5	4	4	1	1	4	3
1527983	2	63	6	5	4	4	1	1	4	3
1527999	1	80	6	5	1	2	9	5	1	1
1528243	1	75	6	5	1	2	9	5	1	1
1528245	1	65	6	5	3	3	3	1	3	2
1528248	2	72	6	5	1	1	9	5	1	1
1599031	1	72	6	5	2	3	9	5	1	1
1599044	2	73	6	5	4	2	9	5	1	1
1599051	1	60	6	5	4	4	1	1	1	1
1599053	2	60	6	5	4	4	3	1	1	1
1599069	2	64	6	5	1	1	9	5	1	1
1599070	1	60	6	5	2	3	9	5	1	1
1599100	2	63	6	5	1	2	9	5	1	1
1599105	1	60	6	5	1	3	6	3	1	1
1599109	1	66	6	5	2	4	9	5	1	1
1599114	2	67	6	5	2	3	9	5	1	1

1599119	1	69	6	5	1	2	9	5	1	1
1599120	2	73	6	5	1	2	9	5	1	1
1599131	2	62	6	5	3	3	3	1	1	1
1599132	1	70	6	5	1	2	9	5	1	1
1599141	2	67	6	5	2	2	9	5	1	1
1599145	1	84	6	5	1	1	9	5	1	1
1599178	2	61	6	5	2	3	9	5	1	1
1599195	1	79	6	5	1	4	9	5	1	1
1599198	1	71	6	5	1	2	9	5	1	1
1599210	2	64	6	5	2	1	9	5	1	1
1599213	1	62	6	5	3	1	9	5	1	1
1599234	2	71	6	5	1	1	9	5	1	1
1599235	1	67	6	5	1	2	9	5	1	1
1599247	1	65	6	5	3	3	9	5	1	1
1599256	2	70	6	5	2	1	9	5	1	1
1599261	2	70	6	5	1	2	9	5	1	1
1599262	1	68	6	5	1	2	9	5	1	1
1599289	1	67	6	5	3	3	9	5	1	1
1599303	2	68	6	5	1	2	9	5	1	1
1599306	1	60	6	5	2	4	4	2	1	1
1599314	1	65	6	5	2	4	6	3	1	1
1599351	1	68	6	5	2	1	9	5	1	1
1599353	2	64	6	5	3	3	9	5	1	1
1599365	1	67	6	5	1	2	9	5	1	1
1599373	2	63	6	5	1	3	9	5	1	1
1599378	2	64	6	5	2	2	9	5	1	1
1599391	2	65	6	5	2	3	9	5	1	1
1599395	1	69	6	5	2	4	9	5	1	1
1599425	2	67	6	5	1	1	9	5	1	1
1599428	1	67	6	5	2	1	10	7	1	1
1599438	2	66	6	5	2	3	9	5	1	1
1599439	1	64	6	5	3	4	9	5	1	1
1599452	1	62	6	5	2	3	9	5	2	2
1599464	1	67	6	5	2	3	9	5	2	2
1599467	2	64	6	5	2	2	4	2	2	2
1599473	1	73	6	5	3	3	9	5	2	2
1599474	2	68	6	5	3	3	9	5	2	2
1599486	2	60	6	5	3	4	7	4	2	2
1599487	1	60	6	5	2	4	4	2	2	2
1599497	2	66	6	5	4	2	9	5	2	2
1599500	2	74	6	5	1	1	9	5	2	2
1599511	2	75	6	5	1	1	9	5	2	2
1599521	2	70	6	5	3	1	9	5	2	2
1599522	1	70	6	5	3	1	9	5	2	2
1599523	2	71	6	5	3	3	9	5	2	2
1599535	2	66	6	5	1	2	9	5	2	2
1599546	2	67	6	5	2	2	9	5	2	2
1599547	1	63	6	5	3	3	4	2	2	2
1599550	2	60	6	5	3	2	9	5	2	2
1599558	2	63	6	5	3	3	9	5	2	2
1599561	2	60	6	5	3	1	4	2	2	2
1599562	1	67	6	5	2	1	4	2	2	2
1599573	2	68	6	5	3	3	9	5	2	2
1599574	1	70	6	5	1	2	9	5	2	2
1599575	2	65	6	5	1	1	9	5	2	2
1599596	2	60	6	5	2	3	3	1	2	2
1599597	1	62	6	5	2	2	9	5	2	2
1599598	2	62	6	5	3	4	3	1	2	2
1599608	2	61	6	5	3	4	3	1	2	2
1599610	1	64	6	5	3	3	4	2	2	2
1599611	2	60	6	5	1	1	9	5	2	2
1599616	2	63	6	5	2	2	9	5	2	2
1599624	2	70	6	5	2	1	9	5	2	2
1599634	1	77	6	5	1	1	9	5	2	2
1599635	2	67	6	5	2	2	9	5	2	2
1599648	2	61	6	5	2	1	9	5	2	2
1599651	2	77	6	5	4	3	9	5	2	2
1599652	1	62	6	5	3	2	9	5	2	2
1599673	2	60	6	5	1	3	9	5	2	2
1599688	2	75	6	5	4	2	10	7	2	2
1599689	1	60	6	5	2	4	4	2	2	2
1599690	2	66	6	5	3	3	10	7	2	2
1599701	2	68	6	5	3	4	9	5	3	2
1599702	1	61	6	5	3	4	4	2	3	2
1599711	2	60	6	5	1	1	10	7	3	2
1599720	2	74	6	5	3	3	9	5	3	2
1599726	2	60	6	5	2	1	9	5	3	2
1599729	1	60	6	5	2	2	9	5	3	2
1599734	2	73	6	5	2	3	9	5	3	2
1599737	2	67	6	5	2	3	9	5	3	2
1599740	1	65	6	5	3	3	9	5	3	2
1599741	2	64	6	5	2	1	9	5	3	2
1599749	2	60	6	5	3	1	4	2	3	2
1599752	2	79	6	5	2	1	9	5	3	2
1599766	2	61	6	5	2	2	4	2	3	2
1599774	1	79	6	5	1	3	9	5	3	2
1599775	2	73	6	5	1	2	9	5	3	2
1599784	1	64	6	5	1	2	1	1	3	2
1599786	2	72	6	5	3	4	9	5	3	2
1599787	1	62	6	5	3	4	3	1	3	2
1599796	2	62	6	5	2	3	9	5	3	2
1599797	1	76	6	5	1	2	9	5	3	2
1599798	2	71	6	5	1	4	9	5	3	2
1599803	2	60	6	5	3	2	3	1	3	2
1599804	1	60	6	5	2	1	9	5	3	2
1599822	1	80	6	5	2	2	9	5	3	2
1599823	2	60	6	5	3	4	9	5	3	2
1599827	2	62	6	5	2	2	9	5	3	2
1599828	1	67	6	5	2	2	9	5	3	2
1599850	2	66	6	5	3	2	9	5	3	2
1599855	2	68	6	5	3	1	9	5	3	2
1599868	2	64	6	5	3	3	9	5	3	2
1599869	1	65	6	5	3	4	9	5	3	2
1599870	2	64	6	5	4	4	9	5	3	2
1599882	1	81	6	5	2	3	9	5	3	2
1599891	2	69	6	5	3	4	9	5	3	2
1599892	1	76	6	5	2	1	9	5	3	2
1599893	2	65	6	5	1	4	9	5	3	2
1599896	2	62	6	5	1	3	9	5	3	2
1599906	2	78	6	5	2	1	9	5	3	2
1599921	2	65	6	5	3	1	9	5	3	2

1599923	1	68	6	5	3	3	9	5	3	2
1599933	1	68	6	5	3	4	1	1	3	2
1599936	2	62	6	5	3	3	9	5	3	2
1599941	2	60	6	5	4	4	1	1	3	2
1599945	2	60	6	5	3	2	9	5	3	2
1599958	2	62	6	5	3	3	9	5	3	2
1599960	2	71	6	5	1	1	9	5	3	2
1599969	1	70	6	5	3	3	9	5	3	2
1599970	2	60	6	5	3	1	9	5	3	2
1599981	2	62	6	5	2	2	4	2	4	3
1599998	2	70	6	5	3	3	9	5	4	3
1599999	1	60	6	5	3	3	4	2	4	3
1600000	2	73	6	5	3	1	9	5	4	3
1600026	2	62	6	5	2	2	9	5	4	3
1600034	1	73	6	5	2	3	9	5	4	3
1600044	1	61	6	5	3	3	3	1	4	3
1600045	2	61	6	5	3	2	3	1	4	3
1600046	2	65	6	5	3	3	9	5	4	3
1600048	1	67	6	5	2	2	9	5	4	3
1600066	1	66	6	5	3	3	3	1	4	3
1600067	2	66	6	5	3	3	3	1	4	3
1600076	1	60	6	5	2	2	10	7	4	3
1600080	2	63	6	5	2	4	4	2	4	3
1600094	1	72	6	5	2	1	9	5	4	3
1601802	2	72	6	5	3	1	9	5	2	2
1601854	2	63	6	5	2	1	9	5	1	1
1601866	2	60	6	5	3	4	1	1	2	2
1601908	1	72	6	5	1	2	9	5	1	1
1601910	2	72	6	5	1	1	9	5	1	1
1672931	1	70	6	5	1	3	9	5	1	1
1672937	2	63	6	5	2	3	9	5	1	1
1672938	1	71	6	5	1	4	9	5	1	1
1672956	2	63	6	5	1	1	9	5	1	1
1672959	2	63	6	5	2	3	9	5	1	1
1672972	2	67	6	5	1	1	9	5	1	1
1672973	1	61	6	5	1	1	9	5	1	1
1672974	2	75	6	5	2	1	9	5	1	1
1672978	1	75	6	5	1	1	9	5	1	1
1672984	2	65	6	5	2	1	9	5	1	1
1672985	2	68	6	5	1	1	9	5	1	1
1672987	1	70	6	5	3	3	9	5	1	1
1672993	2	60	6	5	2	1	9	5	1	1
1672998	1	60	6	5	3	3	4	2	1	1
1673003	2	63	6	5	1	3	9	5	1	1
1673012	2	67	6	5	3	3	9	5	1	1
1673035	1	62	6	5	2	1	9	5	1	1
1673038	2	66	6	5	2	3	9	5	1	1
1673049	2	80	6	5	1	1	9	5	1	1
1673052	1	81	6	5	1	2	9	5	1	1
1673060	1	70	6	5	1	2	9	5	1	1
1673063	2	61	6	5	4	4	9	5	1	1
1673082	1	69	6	5	2	1	9	5	1	1
1673083	2	60	6	5	3	3	9	5	1	1
1673084	2	63	6	5	2	3	9	5	1	1
1673085	1	67	6	5	2	3	9	5	1	1
1673090	1	60	6	5	1	3	10	7	1	1
1673103	2	73	6	5	2	4	9	5	1	1
1673107	1	60	6	5	2	4	6	3	1	1
1673108	2	65	6	5	1	4	6	3	1	1
1673109	2	67	6	5	2	3	9	5	1	1
1673110	1	74	6	5	2	2	9	5	1	1
1673114	2	60	6	5	3	3	4	2	1	1
1673120	1	70	6	5	2	4	9	5	1	1
1673123	2	64	6	5	2	4	9	5	1	1
1673136	1	62	6	5	2	1	9	5	1	1
1673149	2	73	6	5	3	4	9	5	1	1
1673150	1	77	6	5	2	4	9	5	1	1
1673172	1	75	6	5	1	4	9	5	1	1
1673173	2	76	6	5	1	1	9	5	1	1
1673182	1	66	6	5	2	1	9	5	1	1
1673184	2	71	6	5	1	3	9	5	1	1
1673186	1	74	6	5	2	1	9	5	1	1
1673187	2	78	6	5	1	1	9	5	1	1
1673198	2	63	6	5	3	3	9	5	1	1
1673200	2	61	6	5	3	4	3	1	1	1
1673202	1	63	6	5	2	3	4	2	1	1
1673228	1	60	6	5	2	1	9	5	1	1
1673233	2	68	6	5	2	3	9	5	1	1
1673239	1	64	6	5	2	3	9	5	1	1
1673243	2	60	6	5	3	3	3	1	1	1
1673244	2	63	6	5	2	3	9	5	1	1
1673259	2	61	6	5	1	4	9	5	1	1
1673266	2	68	6	5	3	4	9	5	1	1
1673281	2	61	6	5	4	1	9	5	1	1
1673282	1	70	6	5	4	3	9	5	1	1
1673285	1	63	6	5	3	4	1	1	1	1
1673304	2	68	6	5	2	3	9	5	1	1
1673317	1	79	6	5	2	3	9	5	1	1
1673325	2	68	6	5	1	1	9	5	1	1
1673326	1	62	6	5	2	2	9	5	1	1
1673339	2	65	6	5	3	1	9	5	2	2
1673362	1	71	6	5	3	2	9	5	2	2
1673363	2	69	6	5	2	3	9	5	2	2
1673376	1	60	6	5	1	2	9	5	2	2
1673384	2	76	6	5	1	1	9	5	2	2
1673395	2	60	6	5	3	1	9	5	2	2
1673397	1	76	6	5	1	1	9	5	2	2
1673398	2	70	6	5	2	1	9	5	2	2
1673409	2	66	6	5	1	4	9	5	2	2
1673431	2	70	6	5	2	3	9	5	2	2
1673432	1	68	6	5	2	3	9	5	2	2
1673434	2	62	6	5	2	3	9	5	2	2
1673437	1	63	6	5	2	4	9	5	2	2
1673449	2	73	6	5	2	4	9	5	2	2
1673455	1	63	6	5	2	1	9	5	2	2
1673458	2	61	6	5	2	2	9	5	2	2
1673461	2	60	6	5	2	4	4	2	2	2
1673473	2	60	6	5	3	4	3	1	2	2
1673495	1	68	6	5	3	1	9	5	2	2
1673505	2	61	6	5	3	4	4	2	2	2
1673506	1	61	6	5	1	1	9	5	2	2

1673514	2	61	6	5	2	3	9	5	2	2
1673523	2	63	6	5	3	3	9	5	2	2
1673525	2	60	6	5	3	3	4	2	2	2
1673529	1	63	6	5	2	3	4	2	2	2
1673532	2	60	6	5	2	3	4	2	2	2
1673536	1	68	6	5	2	4	9	5	2	2
1673537	2	67	6	5	1	4	9	5	2	2
1673545	2	60	6	5	1	1	9	5	2	2
1673546	1	65	6	5	2	1	9	5	2	2
1673557	2	76	6	5	2	1	9	5	2	2
1673559	2	63	6	5	2	2	9	5	2	2
1673568	2	78	6	5	1	3	9	5	2	2
1673569	1	67	6	5	3	3	9	5	2	2
1673576	1	68	6	5	3	3	9	5	2	2
1673577	2	68	6	5	1	1	9	5	2	2
1673591	2	68	6	5	2	4	9	5	2	2
1673593	2	67	6	5	3	1	9	5	2	2
1673597	1	66	6	5	2	1	9	5	2	2
1673598	2	65	6	5	3	3	9	5	2	2
1673599	2	63	6	5	2	2	9	5	3	2
1673600	1	60	6	5	2	2	3	1	3	2
1673624	2	78	6	5	1	1	9	5	3	2
1673627	1	67	6	5	1	1	9	5	3	2
1673636	2	68	6	5	2	3	9	5	3	2
1673646	2	74	6	5	2	3	9	5	3	2
1673650	1	72	6	5	2	3	9	5	3	2
1673658	2	61	6	5	2	3	9	5	3	2
1673665	1	62	6	5	2	2	9	5	3	2
1673671	2	70	6	5	3	4	9	5	3	2
1673673	2	68	6	5	1	1	9	5	3	2
1673694	2	77	6	5	1	3	9	5	3	2
1673695	1	68	6	5	2	3	9	5	3	2
1673696	2	81	6	5	1	4	9	5	3	2
1673702	2	78	6	5	1	1	9	5	3	2
1673703	1	61	6	5	2	2	4	2	3	2
1673709	2	80	6	5	3	4	9	5	3	2
1673712	1	79	6	5	1	4	9	5	3	2
1673720	2	68	6	5	2	1	9	5	3	2
1673727	2	81	6	5	3	4	9	5	3	2
1673728	1	64	6	5	2	1	10	7	3	2
1673729	2	68	6	5	1	2	9	5	3	2
1673742	2	75	6	5	3	1	9	5	3	2
1673750	1	60	6	5	1	2	9	5	3	2
1673759	1	63	6	5	1	1	9	5	3	2
1673760	2	60	6	5	2	3	10	7	3	2
1673766	2	88	6	5	3	4	9	5	3	2
1673767	1	60	6	5	3	4	3	1	3	2
1673768	2	64	6	5	3	4	3	1	3	2
1673789	2	72	6	5	1	4	9	5	3	2
1673790	1	66	6	5	2	4	9	5	3	2
1673791	2	62	6	5	2	4	9	5	3	2
1673795	1	61	6	5	4	4	1	1	3	2
1673804	2	85	6	5	4	2	9	5	3	2
1673807	1	65	6	5	1	1	9	5	3	2
1673808	2	66	6	5	3	4	9	5	3	2
1673812	1	65	6	5	1	4	9	5	3	2
1673813	2	62	6	5	2	3	9	5	3	2
1673819	2	68	6	5	2	2	9	5	3	2
1673820	1	64	6	5	3	3	4	2	3	2
1673821	2	64	6	5	3	4	9	5	3	2
1673828	2	62	6	5	4	4	1	1	3	2
1673834	2	61	6	5	2	1	9	5	3	2
1673835	1	61	6	5	2	3	4	2	3	2
1673839	2	68	6	5	2	4	9	5	3	2
1673840	1	70	6	5	2	2	9	5	3	2
1673843	2	60	6	5	2	1	4	2	3	2
1673852	1	77	6	5	3	2	9	5	3	2
1673856	2	61	6	5	3	1	9	5	3	2
1673857	1	61	6	5	3	2	9	5	3	2
1673858	2	61	6	5	3	1	9	5	3	2
1673867	1	68	6	5	4	1	9	5	3	2
1673868	2	62	6	5	4	4	9	5	3	2
1673869	2	61	6	5	3	3	9	5	4	3
1673880	1	73	6	5	4	2	9	5	4	3
1673881	2	79	6	5	3	1	9	5	4	3
1673889	1	60	6	5	3	1	3	1	4	3
1673903	2	63	6	5	3	1	9	5	4	3
1673909	2	63	6	5	3	3	3	1	4	3
1673915	1	71	6	5	2	3	9	5	4	3
1673923	1	63	6	5	3	4	9	5	4	3
1673924	2	62	6	5	3	3	9	5	4	3
1673931	2	63	6	5	1	1	9	5	4	3
1673932	1	62	6	5	2	2	3	1	4	3
1673933	2	60	6	5	3	3	3	1	4	3
1673942	1	72	6	5	2	3	9	5	4	3
1673943	2	74	6	5	2	2	9	5	4	3
1673944	2	62	6	5	3	1	3	1	4	3
1673946	1	65	6	5	3	3	3	1	4	3
1673965	2	75	6	5	2	1	9	5	4	3
1673974	1	67	6	5	1	3	9	5	4	3
1673978	2	61	6	5	3	3	9	5	4	3
1673982	1	64	6	5	4	4	2	1	4	3
1673992	1	61	6	5	2	3	4	2	4	3
1674642	2	66	6	5	2	3	9	5	1	1
1674643	1	74	6	5	3	4	9	5	1	1
1674649	1	70	6	5	1	1	9	5	3	2
1674650	2	62	6	5	2	4	9	5	3	2
1674659	2	87	6	5	1	1	9	5	2	2
1674671	1	63	6	5	2	1	9	5	3	2
1674700	2	67	6	5	3	1	9	5	1	1
1674706	2	69	6	5	1	2	9	5	1	1
